# Shuangshen granules enhance anti-PD1 therapy effectiveness in lung adenocarcinoma by modulating myeloid-derived suppressor cell-induced T cell exhaustion

**DOI:** 10.1186/s13020-026-01391-3

**Published:** 2026-05-06

**Authors:** Zhong-ning He, Qi Huang, Yi Li, Jia-qi Hu, Tong-tong Liu, Yu-wei Zhao, Xiao-ling Ren, Shu-lin He, Yue Li, Bo-lun Shi, Rui Liu, Qiu-jun Guo, Xing Zhang, Zhan Shi, Jie He, Run-zhi Qi, Bao-jin Hua

**Affiliations:** 1 Department of Oncology, Academy of Chinese Medical Sciences, Guang’anmen Hospital, China , Beijing, 100053 China; 2 Institute of Basic Research in Clinical Medicine, China, Academy of Chinese Medical Science , Beijing, 100700 China; 3 Graduate School, Beijing University of Chinese Medicine, Beijing, 100000 China; 4 Women and Children’s Hospital, School of Medicine, Xiamen University, Xiamen, 361000 China

**Keywords:** Shuangshen granules, Lung adenocarcinoma, Myeloid-derived suppressor cells, Immunotherapy, T cell exhaustion

## Abstract

**Background:**

Worldwide, lung cancer is the most common cause of cancer-related deaths. Molecular targeted therapies and immunotherapies for non-small-cell lung cancer (NSCLC) have improved outcomes markedly over the past two decades. However, the vast majority of advanced NSCLCs become resistant to current treatments and eventually progress. A traditional Chinese medicine (TCM) formula of Shuangshen granules (SSG) has demonstrated potential in alleviating cancer side effects and improving survival rate. Despite clinical evidence supporting its benefit, there is still insufficient understanding of the active compounds in SSG and their underlying mechanisms, which limits its broader clinical application.

**Methods:**

Lewis lung carcinoma (LLC) tumor-bearing mouse model was established to assess the efficacy of combined SSG and anti-PD-1 therapy in vivo, and myeloid-derived suppressor cells (MDSC) and CD8^+^T cells were isolated for in vitro co-culture experiments, while pathological examination was conducted using hematoxylin and eosin (HE). The expression of PD-1, TIM-3, CTLA-4, LAG-3, Arg-1, IDO, iNOS, PD-L1 and Gal-9 was detected using immunohistochemistry (IHC), immunofluorescence, and flow cytometry and Western blotting. The expression of IL-2, TNF-α and IFN-γ were detected by reverse transcription-quantitative polymerase chain reaction (qPCR). Concentrations of IL-10 and TGF-β were measured by enzyme-linked immunosorbent assay (ELISA). Network pharmacology and molecular docking were utilized to screen for potential therapeutic targets and intervening signaling pathways of SSG in lung adenocarcinoma (LUAD). The predictions derived from this approach were further verified using Western blotting.

**Results:**

In vivo experiments using LLC xenograft mice demonstrated that SSG suppressed tumor growth in a dose-dependent manner, with high-dose SSG showing optimal efficacy in inhibiting tumor angiogenesis and cell proliferation. SSG enhances anti-tumor immunity by reducing T cell exhaustion and MDSC-mediated immunosuppression, with SSG + anti-PD-1 combination therapy synergistically optimizing the tumor immune microenvironment. Network pharmacology analysis revealed 5 hub targets (IL2, STAT3, HSP90AA1, LGALS3, and FGF2) associated with immune, LUAD, and active ingredients of SSG, with significant enrichment in the PI3K-Akt pathway. Compared with the control group, the protein expression levels of p-PI3K and p-Akt in the SSG group were significantly down-regulated, indicating that the PI3K-Akt pathway may be inhibited.

**Conclusions:**

SSG could dose-dependently inhibit LLC tumor growth in mice and exert antitumor effects by alleviating T-cell exhaustion and MDSC-mediated immunosuppression. Notably, IL2, STAT3, HSP90AA1, LGALS3 and FGF2, as potential targets of SSG, were significantly enriched in the PI3K-Akt pathway, which provide a novel perspective for the treatment of LUAD.

**Supplementary Information:**

The online version contains supplementary material available at 10.1186/s13020-026-01391-3.

## Introduction

Lung cancer remains the most commonly diagnosed malignancy and the leading cause of cancer-related death worldwide. In 2022, it accounted for nearly 2.5 million new cases and more than 1.8 million deaths, representing approximately one in eight of all cancer diagnoses (12.4%) and almost one in five cancer deaths (18.7%) globally. Despite advances in surgery, radiotherapy, chemotherapy, and targeted therapies, the overall prognosis of lung cancer patients remains poor, underscoring the urgent need for more effective treatment strategies [1]. In recent years, immune checkpoint inhibitors (ICIs), particularly antibodies targeting the programmed cell death 1 (PD-1)/programmed cell death ligand 1 (PD-L1) axis, have revolutionized the treatment landscape of advanced non-small cell lung cancer (NSCLC) [2]. These agents can restore antitumor T cell activity and prolong survival in a subset of patients [2]. However, the clinical benefit of PD-1/PD-L1 blockade is limited, as only a fraction of patients achieves durable responses. A major obstacle to immunotherapy efficacy is the immunosuppressive tumor microenvironment (TME), which promotes T cell dysfunction and exhaustion [3, 4].

T cell exhaustion is a state of T cell dysfunction that arises during chronic infection and tumor progression. It is characterized by the upregulation and co-expression of multiple inhibitory receptors and a concomitant reduction in the cytotoxic activity of effector T cells [5]. This exhausted state weakens the anti-tumor immune function of T cells and also limits the effectiveness of ICIs and other immunotherapies [6]. Among the key regulators of this immunosuppressive milieu are myeloid-derived suppressor cells (MDSCs), a heterogeneous population of immature myeloid cells that expand in cancer and inhibit antitumor immunity [7]. MDSCs suppress CD8⁺ T cell function through the expression of arginase-1, inducible nitric oxide synthase, and immunoregulatory cytokines, while also driving the upregulation of multiple exhaustion markers, including PD-1, TIM-3, and LAG-3. This culminates in the impaired function of tissue-resident memory T cells (TRMs), which are critical for sustained local immune surveillance and antitumor responses [8–10]. Therefore, targeting MDSCs within the immunosuppressive tumor microenvironment is considered a promising approach to reverse T cell exhaustion [11, 12].

Traditional Chinese medicine (TCM) has long been used as an adjuvant approach in oncology, and accumulating evidence suggests that specific herbal formulations may modulate immune responses and improve therapeutic outcomes due to its complex composition and multi-target mechanisms. Feiyuping ointment, which contains Shuangshen granules (SSG) as the primary component, is commonly used in clinical practice for NSCLC patients and has shown promise as an effective TCM therapy. Studies have demonstrated that SSG can significantly improve quality of life and extend survival in patients with NSCLC [13, 14]. SSG is a formulation developed at Guang’anmen Hospital (patent No. 201310091864.4) composed of Panax quinquefolium L., Panax notoginseng, and Cordyceps sinensis, and it has undergone extensive pharmacological and toxicological testing. For nearly a decade, SSG has shown notable therapeutic benefits in the clinical management of lung adenocarcinoma (LUAD). Specifically, 20(S)-Protopanaxadiol (PPD), as a glycoside saponin and ginsenoside isolated from Panax quinquefolium L, triggers mitochondrial-mediated caspase dependent apoptosis by down-regulating the PI3K/Akt signaling pathway in LUAD A549 cells [15]. Trilinolein, an active ingredient isolated from Panax notoginseng, exerts dose- and time-dependent growth inhibition and apoptosis induction in A549 cells, with the Bcl-2 family and caspase-3 serving as core regulators linked to cytochrome c release and Akt signaling dephosphorylation [16]. Moreover, hirsutella sinensis fungus (HSF), an artificial substitute for Cordyceps sinensis, inhibits lung cancer progression by promoting macrophage M1 polarization and activating CD8⁺ T cells via CCRL2 [17]. Collectively, these complementary anti-tumor mechanisms of SSG’s key components lay a critical foundation for the formulation’s robust therapeutic efficacy against LUAD. This was further supported by previous evidence demonstrating that SSG inhibits the differentiation of myeloid cells into MDSCs, thus limiting lung cancer metastasis [18]. It has shown potential in restoring immune balance and enhancing the efficacy of conventional therapies. Nevertheless, its precise effects on MDSC-mediated immunosuppression and TRM cell exhaustion in the context of PD-1 blockade remain unclear.

Therefore, in this study, we investigated the impact of SSG on tumor growth, immune cell composition, and functional cytokine expression in a murine model of LUAD. We further explored the potential of SSG to alleviate MDSC-induced T cell exhaustion and augment the efficacy of anti-PD-1 therapy, and investigated and verified the possible mechanisms by which SSG may play a role, providing new mechanism insights and translation significance for the combination of traditional Chinese medicine and modern immunotherapy.

## Materials and methods

### Materials, reagents, and cell lines

Panax quinquefolium L. (Batch No. 23011602), Panax notoginseng (Batch No. 21030903), and Cordyceps sinensis (Batch No. 230360511) were obtained from Guang’anmen Hospital of the China Academy of Chinese Medical Sciences (Beijing, China) (see Table 1 for details). Ginsenoside Rg1 (No. 110703–202235), ginsenoside Rb1 (No. 110704–202331), ginsenoside Rd (No. 111818–202,104), and notoginsenoside R1 (No. 110745–202322) were obtained from the China Institute of Food and Drug Control (Beijing, China). RIPA lysis buffer was from Promega (Madison, WI, USA), RPMI-1640 medium was from Solarbio (Beijing, China), and DMEM and fetal bovine serum (FBS) were from HyClone (Logan, UT, USA).

**Table 1:** The information pertaining to the Chinese medicines found in the SSG-derived herb

Chinese Name	Latin name	Family	Place of origin (province)	Used part	Major effective compound in modern pharmacology study
Xi Yang Shen	*Panax quinquefolium*	Araliaceae	Ji Lin	Rhizome	Ginsenosides
San Qi	*Panax notoginseng*	Araliaceae	Yun Nan	Rhizome	Notoginsenosides
Dong Chong Xia Cao	*Cordyceps sinensis*	Clavicipitaceae	Tibet	Stroma–larva complex^a^	Nucleosides, polysaccharides, and cordycepin

^a^ The dried complex of the stromata of the fungus and the dead bodies of the larvae of insects in the family Bat Moths

Primary antibodies against Arg-1 (arginase-1, #93,668), IDO (#51,851), PD-L1 (#64,988), TIM-3 (#83,882), CTLA-4 (#53,560), LAG-3 (#80,282), and β-actin (#3700) were purchased from Cell Signaling Technology (CST, Boston, MA, USA). Antibodies against iNOS (ab178945), Galectin-9 (Gal-9, ab69630), PD-1 (ab214421), and BTLA (ab216505) were from Abcam (Cambridge, UK). An anti-mouse PD-1 monoclonal antibody (clone RMP1-14, BE0273) for in vivo use was obtained from BioXcell (West Lebanon, NH, USA). Primary antibodies used in Western blot analysis, including GAPDH Rabbit pAb (AC001), IL2 Rabbit mAb (A22200), Pan-Akt Rabbit mAb (A18675), Phospho-AKT-S473 Rabbit mAb (AP0637), PIK3CG Rabbit pAb (A6688), STAT3 Rabbit mAb (A19566), and Phospho-PI3 Kinase p55-Y199 Rabbit mAb (AP1463), were purchased from Abclonal (Wuhan, China). Hsp90 alpha antibody (AB3632) was obtained from ABWAYS (Shanghai, China); Galectin-3 polyclonal antibody (14,979–1-AP) and FGF2 polyclonal antibody (30,333–1-AP) were from Proteintech Group, Inc (Wuhan, China). The horseradish peroxidase (HRP)-conjugated goat anti-rabbit IgG (H + L) secondary antibody (31,460) was purchased from Invitrogen (Shanghai, China). Fluorophore-conjugated antibodies for flow cytometry were purchased from BioLegend (San Diego, CA, USA), including FITC–anti-CD11b (Cat# 101,205), PerCP/Cyanine5.5–anti-Gr-1 (Cat#108,425), PE–anti-PD-L1 (Cat#155,403), PE/Cyanine7–anti-Gal-9 (Cat#137,913), FITC–anti-CD3 (Cat#100,203), PerCP/Cyanine5.5–anti-CD8a (Cat#100,733), PE–anti-PD-1 (Cat#114,117), and Brilliant Violet 605–anti-TIM-3 (Cat# 119,721). ELISA kits for IL-10, TNF-α, IL-2, IFN-γ, and TGF-β were from NeoBioscience (Shenzhen, China). Recombinant GM-CSF (Cat#315–03) and IL-6 (Cat#216–16) were obtained from PeproTech (Rocky Hill, NJ, USA). The Lewis lung carcinoma (LLC) cell line was obtained from the National Infrastructure of Cell Line Resource (Beijing, China) and maintained in DMEM supplemented with 10% FBS at 37 °C in a humidified 5% CO_2_ incubator.

### Establishment of the tumor-bearing mouse model and SSG treatment

SSG was prepared from authenticated raw herbal materials (Panax quinquefolius, Panax notoginseng, and Cordyceps sinensis) by pulverizing the herbs and mixing them in a 1:6:1 (w/w/w) ratio. Quality control for the raw herbs and the final formulation followed protocols from our previous studies [18, 19]. Male C57BL/6 J mice (4–5 weeks old) were acclimated for 7 days prior to tumor implantation. To establish the tumor model, LLC cells (5 × 10^^^6 in 100 μL PBS) were injected subcutaneously into the left flank of each mouse [20]. To determine the optimal SSG dosage, tumor-bearing mice were randomly divided into four groups (n = 5 per group): Control, low-dose SSG (SSG-L), medium-dose SSG (SSG-M), and high-dose SSG (SSG-H). The SSG-L dose was based on the human equivalent dose of the herbal formula (~ 6 g per 70 kg adult), which corresponds to approximately 18.0 mg/day for a ~ 20 g mouse using a body surface area conversion factor (mouse:human ratio of 9.01). SSG-M and SSG-H were set at two times (36.0 mg/day) and three times (54.0 mg/day) the SSG-L dose, respectively. SSG was administered orally once daily for 21 days, while the Control group received an equal volume of water. After 21 days of drug intervention, 0.4% pentobarbital sodium at a dose of 30 mg/kg was injected intraperitoneally to anesthetize the mice. Peripheral blood, spleen, and tumor tissues from each group of mice were collected for subsequent experiments. Based on the extent of tumor inhibition observed, the high dose of SSG was selected as the optimal dose for subsequent experiments.

**Fig. 1:**
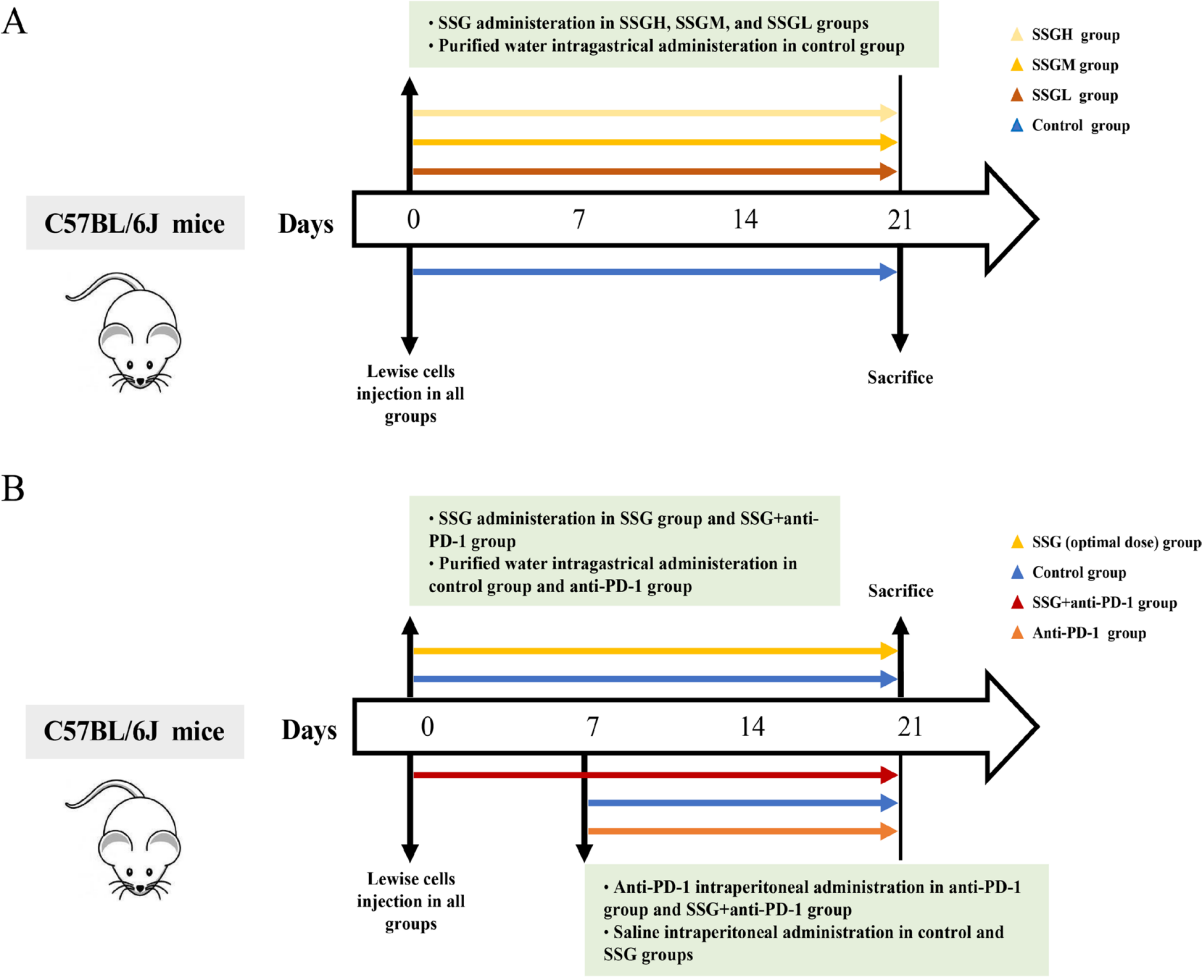
Timeline of the animal experiments. (**A**) Timeline of the SSG dose-optimization experiment. (**B**) Timeline of the treatment schedule for control, SSG, anti-PD-1, and combination therapy groups in the main in vivo study

For the combination therapy experiment, another set of LLC tumor-bearing mice were randomly assigned to four groups (n = 8 per group): Control, SSG alone, anti-PD-1 alone, and SSG + anti-PD-1. Mice in the SSG group received the optimal dose of SSG orally each day for 21 days. Mice in the anti-PD-1 group received intraperitoneal injections of anti-mouse PD-1 antibody (200 µg per mouse, every other day) starting on day 7 post-tumor inoculation, for a total of 7 injections (14 days). Control group mice received oral water and i.p. saline on matched schedules. On day 21, all mice were euthanized, and tumor tissues, peripheral blood, and spleens were collected for further analysis. All animal procedures were approved by the Animal Ethics Committee of Guang’anmen Hospital, China Academy of Chinese Medical Sciences (Approval No. IACUC-GAMH-2022–011). The experimental timeline is illustrated in Fig. 1.

### HE and IHC

Excised tumor tissues were fixed in 4% neutral buffered formalin, embedded in paraffin, and sectioned into 4–6 µm slices. For H&E staining, sections were deparaffinized, rehydrated, and stained with hematoxylin followed by eosin. For IHC, tissue sections underwent antigen retrieval in sodium citrate buffer (pH 6.0) and quenching of endogenous peroxidase activity with 3% H_2_O_2_ in methanol. Sections were then blocked with 5% BSA and incubated with primary antibodies overnight at 4 °C. After washing, HRP-conjugated secondary antibodies were applied, and immunoreactivity was visualized using a DAB chromogen. Sections were counterstained (with hematoxylin), mounted with neutral resin, and examined under a light microscope for imaging and scoring.

### Immunofluorescence

Paraffin-embedded tumor sections were deparaffinized and subjected to antigen retrieval as above. After blocking with 5% BSA for 1 h at room temperature, sections were incubated with primary antibodies against CD3, PD-1, CD11b, or PD-L1. Subsequently, sections were incubated with species-appropriate Alexa Fluor–conjugated secondary antibodies. Nuclei were counterstained with DAPI. Stained sections were observed and imaged using a laser scanning confocal microscope (Leica Microsystems, Wetzlar, Germany).

### Flow cytometry

Single-cell suspensions from mouse spleens or cultured cells were prepared and washed with PBS. Cells were first incubated with an anti-mouse CD16/32 Fc-blocker for 15 min on ice to prevent nonspecific antibody binding. Two antibody staining panels were then applied: one panel (for myeloid-derived cells) included FITC-anti-CD11b, PerCP/Cyanine5.5-anti-Gr-1, PE-anti-PD-L1, and PE/Cyanine7-anti-Gal-9; the other panel (for T cells) included FITC-anti-CD3, PerCP/Cyanine5.5-anti-CD8a, PE-anti-PD-1, and BV605-anti-TIM-3. Staining was performed in the dark at 4 °C for 25 min. After staining, cells were washed (1500 rpm, 5 min) and resuspended in FACS buffer (PBS with 2% FBS). Data acquisition was done on a BD LSR II flow cytometer (BD Biosciences), and subsequent data analysis was conducted using FlowJo v10 software, with Tukey’s multiple comparison test applied for statistical analysis.

### Western blotting

Tumor tissue samples or cultured cells were lysed in ice-cold RIPA buffer containing protease and phosphatase inhibitors (Topscience, Shanghai, China) for 30 min. Lysates were clarified by centrifugation at 12,000 rpm for 15 min at 4 °C, and supernatants were collected. Equal amounts of protein (determined by BCA assay) were separated by SDS-PAGE and transferred onto PVDF membranes. Membranes were blocked with 5% BSA in TBST for 1 h at room temperature and then incubated with primary antibodies at 4 °C overnight. After washing, membranes were probed with HRP-conjugated secondary antibodies (ZSGB-Bio, Beijing, China) for 1 h at room temperature. Immunoreactive bands were detected using enhanced chemiluminescence (ECL) reagents and imaged. Band intensities were quantified using ImageJ software (v1.53a).

### Enzyme-linked immunosorbent assay

Peripheral blood samples and cell culture supernatants were centrifuged at 12,000 rpm for 20 min at 4 °C to obtain cell-free supernatants. The concentrations of IL-10 and TGF-β in these supernatants were measured using specific ELISA kits according to the manufacturers’ protocols. Absorbance at the appropriate wavelength was read using a GloMax Discover microplate reader (Promega, Madison, WI, USA), and cytokine concentrations were calculated from standard curves.

### Reverse transcription-quantitative polymerase chain reaction

Total ribonucleic acid (RNA) was extracted from mouse tumor tissues using a Rapid RNA Purification Kit (ES Science, Shanghai, China). cDNA was synthesized with the RevertAid reverse transcription kit (Yeasen, Shanghai, China), followed by reverse transcription-quantitative polymerase chain reaction on an applied biosystems (Bio-Rad T100, USA) to quantify IL-2, IFN-γ, and TNF-α mRNA levels. Gene expression was normalized using GAPDH as a stably expressed reference gene. Triplicate experiments ensured reproducibility, and primers were designed via primer-basic local alignment search tool (National Center for Biotechnology Information, National Institutes of Health, USA) (Table 2).

**Table 2:** Primer sequences for reverse transcription-quantitative polymerase chain reaction

Gene		Primer sequence (5’–3’)
IL-2	Forward	GGCCAGCTGTGAGTGTTTCTTTGG
	Reverse	CTCGCTTCATCTTCCCTCTTGGG
IFN-γ	Forward	TGAACGCTACACACTGCATCTTGG
	Reverse	CTCCTTTTCCGCTTCCTGAG
TNF-α	Forward	CCCTCACACTCAGATCATCTTCT
	Reverse	GCTACGACGTGGGCTACAG
GAPDH	Forward	GTGGACCTGACCTGCCGTCT
	Reverse	GGAGGAGTGGGTGTCGCTGT

### Preparation of SSG drug-containing serum

Male Wistar rats with initial body weights of (180–200) g received SSG extract (62.5 mg/kg, intragastrically, BID) for 7 days. This dose was based on HED conversion from a 6 g/70 kg human dose (rat:human ratio 6.25). Two hours after the final administration, 3% pentobarbital sodium at a dose of 30 mg/kg was intraperitoneally injected to anesthetize the rats, and blood was collected from the abdominal aorta and refrigerated overnight at 4 °C. Following centrifugation at 3,000 rpm for 15 min, sera were collected and stored at -20 °C. The experimental protocol was approved by the Animal Welfare and Ethics Committee of Guang’anmen Hospital, China Academy of Chinese Medical Sciences and conducted in compliance with the International Council for Laboratory Animal Science guidelines (approval No. IACUC-GAMH-2024–004-SQ).

#### Component analysis for SSG drug-containing serum

The major chemical constituents in SSG-containing serum were analyzed using high-performance liquid chromatography coupled with tandem mass spectrometry (HPLC–MS/MS). The analysis was performed on an ExionLC-20AD HPLC system coupled to an AB Sciex mass spectrometer, following a previously described protocol [21]. Optimized multiple reaction monitoring (MRM) parameters for each ginsenoside are listed in Table 3. Prior to analysis, 100 µL of serum was mixed with 300 µL of methanol–acetonitrile (4:1, v/v), vortexed at 6000 rpm for 5 min, and then centrifuged at 20,000 × g for 10 min at 4 °C. The supernatant was collected and evaporated to dryness under a stream of nitrogen. The residue was re-dissolved in methanol to a concentration of 10 mg/mL. A stock solution of mixed standard compounds (1 mg/mL each in methanol) was prepared and serially diluted to generate calibration curves. LC–MS/MS data were acquired in both positive and negative ion modes over an m/z range of 30–1300, with a scan time of 0.4 s. Data acquisition and analysis were performed using MassLynx v4.1 (Waters Corp. Milford, MA, USA) and its Elemental Composition tool (Waters).

**Table 3:** The optimized HPLC–MS parameters of each component

Component	Quantitative ion pair	Dwell time /ms	Collision energy /eV	Declustering Potential /V	Retention /min
Ginsenoside Rg1	823.2/643.3	20	56	129	9.33
Ginsenoside Rb1	1131.7/789.3	20	75	260	10.71
Ginsenoside Rd	969.7/789.6	20	61	270	11.22
Notoginsenoside R1	955.3/775.4	20	46	135	9.07

#### Isolation of MDSCs and CD8^+^ T cells

Bone marrow cells were harvested from the femurs and tibias of 4–5-week-old C57BL/6 J mice. After red blood cell lysis and washing with PBS, the bone marrow cells were cultured in RPMI-1640 medium containing 10% FBS and supplemented with 100 ng/mL GM-CSF and 100 ng/mL IL-6 to induce the differentiation and expansion of MDSCs. After 4 days of incubation, cells were collected and adjusted to 5 × 10^6 cells/mL. Then, the cells were jointly labeled with anti-CD11b and anti-Gr-1 antibodies and analyzed by flow cytometry to confirm the successful enrichment of MDSCs. When the purity of induced-differentiated MDSCs reached 98%, it met the requirements for subsequent experiments. The Gr-1 antibody recognizes epitopes on both Ly6G and Ly6C, thereby labeling the entire MDSC population [22]. The MDSC-enriched cells were then treated with culture medium containing SSG-containing rat serum for 24 h. After treatment, the MDSCs and their culture supernatants were collected for analysis (flow cytometry, Western blotting, and ELISA) to evaluate changes in their immunosuppressive features.

For CD8^+^ T cell isolation, spleens were collected from 4–5-week-old C57BL/6 J mice. Single-cell suspensions were prepared by grinding spleen tissues through 70 µm cell strainers. After red cell lysis, CD8^+^ T cells were isolated using a magnetic CD8a^+^ T Cell Isolation Kit (Miltenyi Biotec, Bergisch Gladbach, Germany) according to the manufacturer’s instructions. Briefly, splenocytes were incubated with anti-CD8a magnetic microbeads and passed through MS columns in a magnetic field; the enriched CD8^+^T cells were then eluted and verified by flow cytometry for purity. MDSCs and CD8^+^T cells (both pretreated with SSG-containing serum as described above) were co-cultured using a Transwell system to assess functional interactions. CD8^+^T cells were placed in the lower chamber of a Transwell plate, and MDSCs were added to the upper insert (pore size ~ 0.4 µm, which permits exchange of factors but prevents cell migration). The two cell populations were co-cultured for the durations specified in subsequent assays. After co-culture, cells (and conditioned media) were collected for flow cytometry, Western blotting, and ELISA analyses, as described earlier.

#### CFSE proliferation assay

Proliferation of CD8^+^T cells was assessed using a CFSE dilution method. Isolated CD8^+^T cells were labeled with 5 µM CFSE (Carboxyfluorescein diacetate succinimidyl ester; BD Biosciences) in the dark at 37 °C for 15 min. Staining was quenched by adding five volumes of cold RPMI-1640 medium. The cells were washed and then resuspended in RPMI-1640 complete medium containing 200 ng/mL IL-2, 10 µg/mL anti-CD3ε, and 10 µg/mL anti-CD28 to provide costimulatory signals. CFSE-labeled CD8^+^T cells (5 × 10^5) were co-cultured with MDSCs (also pretreated with SSG serum) at a 1:1 ratio in 96-well plates for 72 h. After 72 h, cells were harvested and the dilution of CFSE intensity in CD8^+^T cells was measured by flow cytometry to determine T cell proliferation (a decrease in CFSE fluorescence indicates cell division).

#### Apoptosis assay

CD8^+^T cell apoptosis was evaluated after co-culture with MDSCs using an Annexin V-FITC/PI apoptosis detection kit (BD Biosciences, Cat# 556,547). CD8^+^T cells and MDSCs were co-cultured for 24 h in a Transwell setup (CD8^+^T cells in the lower well, MDSCs in the upper insert). After 24 h, CD8^+^T cells were collected, washed with cold PBS, and resuspended in 500 µL of binding buffer. The cells were then stained with 5 µL of Annexin V–FITC and 5 µL of propidium iodide (PI) and incubated in the dark for 30 min at room temperature. Early and late apoptosis of CD8^+^T cells were analyzed by flow cytometry, with Annexin V^+^PI^−^cells considered early apoptotic and Annexin V^+^PI^+^cells considered late apoptotic.

#### Cell Counting Kit-8

Following enrichment as described in Sect. "Isolation of MDSCs and CD8+ T cells", MDSCs were counted and subsequently seeded into 96-well plates, with the outermost wells filled with PBS to eliminate edge effects, followed by incubation in a cell incubator. The seeding density was optimized according to cell size and growth rate, with three replicate wells per group. SSG-containing serum was mixed with 1640 complete medium to prepare six concentration gradients (5%, 10%, 20%, etc.), and a blank serum group (10% blank rat serum) and a fetal bovine serum (FBS) group (10% FBS) were established as controls. MDSCs were treated with the above media for 24 h, after which 10 µL of CCK-8 solution (Servicebio, G1613-1ML) was added to each well for an additional 1 h of incubation. The absorbance at 450 nm (OD_450_) was measured using a microplate reader, and cell viability was calculated via statistical analysis of the acquired data.

#### Network pharmacological analysis of SSG

The SMILES structures of Ginsenoside Rg1, Ginsenoside Rb1, Ginsenoside Rd, and Notoginsenoside R1 were retrieved from the PubChem database (https://pubchem.ncbi.nlm.nih.gov/), and their corresponding targets were screened in the Swiss Target Prediction (http://www.swisstargetprediction.ch), Targetnet (http://targetnet.scbdd.com/), and SEA (https://sea.bkslab.org/) databases with the threshold set at Prob > 0. Immune-related genes were collected from the ImmPort database (https://www.immport.org/). Meanwhile, to improve data reliability, LUAD targets from OMIM (http://www.omim.org/) and GeneCards (https://www.genecards.org/) were filtered via a two-round median cutoff method based on correlation scores, and the final gene set was generated by taking the union of targets from the two databases.

Predicted targets of compounds were intersected with immune-related genes and LUAD–related genes to identify common targets. The overlapping targets among SSG compounds, immune-related genes, and LUAD-related genes were determined using Venny 2.1.0 and were subsequently used to construct a drug–target protein–protein interaction (PPI) network via the Cytoscape software v3.7.1. Then, the PPI network of common targets was constructed using the STRING database (http://string.embl.de/), with a confidence score of 0.15 and Homo sapiens was selected as the species. The degree algorithm was adopted to sort the genes in the network and screen out the top 5 core targets. Gene Ontology (GO) and Kyoto Encyclopedia of Genes and Genomes (KEGG) pathway analyses were conducted to elucidate the biological functions and pathways associated with the targets [23]. The aforementioned analyses were adjusted for false discovery rate (FDR).

#### Molecular docking study

Molecular docking was carried out to evaluate potential interactions between key active SSG compounds and high-priority protein targets identified from the network analysis. The crystal structures of target proteins were obtained from the uniqort/PDB database and prepared by removing water molecules using PyMOL. Chemical structures of the major active components were sketched and optimized to low-energy three-dimensional conformations using Chem3D. Each compound was then docked to its respective target protein using AutoDock Vina v1.5.6, and the binding pose with the lowest binding free energy was recorded for analysis.

#### Statistical analysis

Normality and variance homogeneity were assessed prior to analysis. For normally distributed parameters with equal variances, data are expressed as mean ± standard deviation and analyzed by parametric one-way analysis of variance (ANOVA), with post-hoc comparisons adjusted via False Discovery Rate test. When normality or variance assumptions were violated, non-normally distributed parameters are presented as median (interquartile range) and analyzed using the Kruskal–Wallis test (non-parametric equivalent of ANOVA), followed by Dunn's post-hoc comparisons. All statistical analyses were performed using GraphPad Prism 8.0 (GraphPad Software, USA), with statistical significance defined as a two-tailed P < 0.05.

## Results

### SSG inhibits tumor growth in a xenograft mouse model

**Fig. 2:**
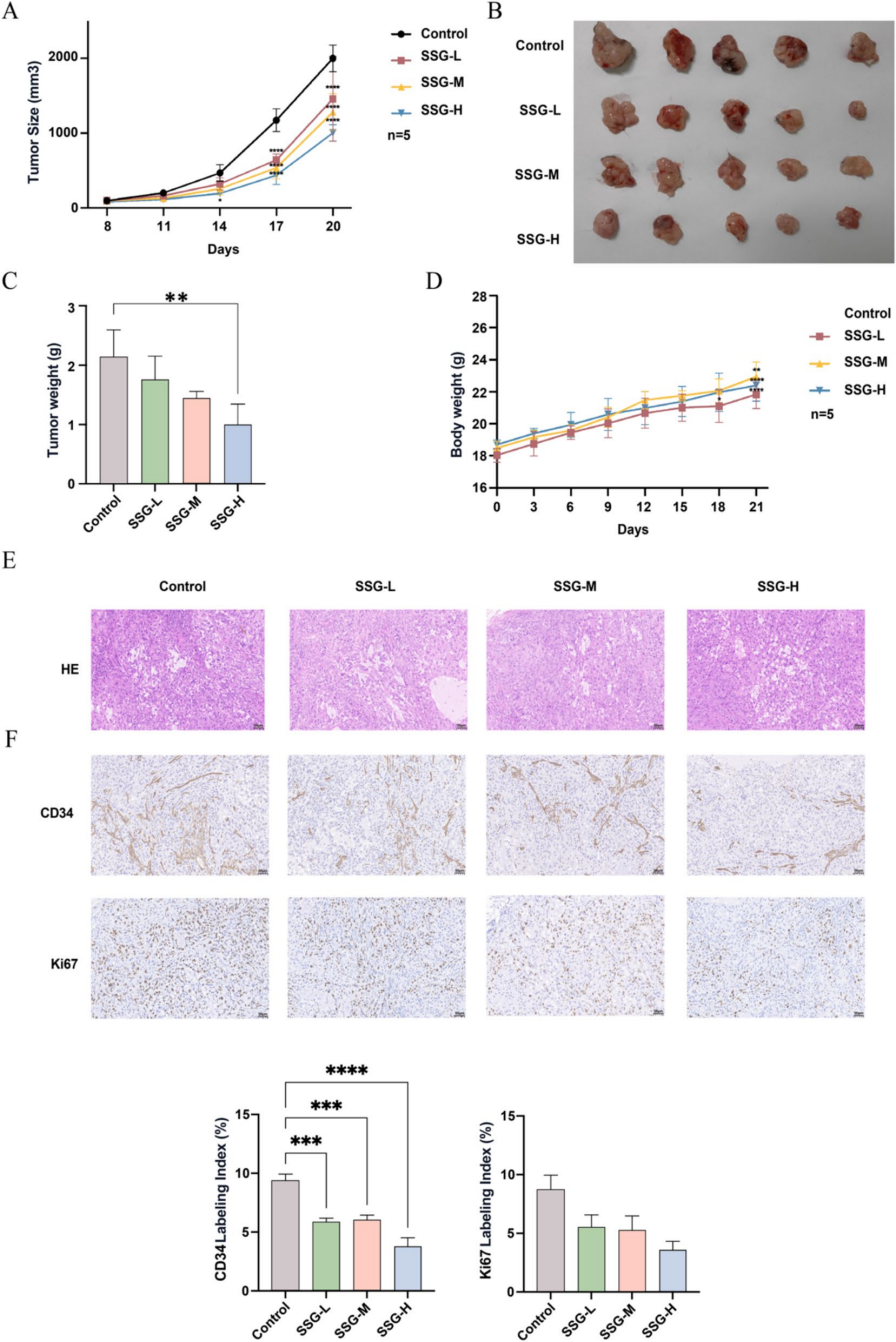
Effects of different SSG doses on tumor growth and proliferation in LLC tumor-bearing mice (n = 5). (**A**) Tumor volume growth curves for Control vs. low (L), medium (M), and high (H) SSG dose groups. (*P < 0.05, ****P < 0.0001 for SSG-H vs Control). (**B**) Photographs of excised tumors from each group at endpoint. (**C**) Tumor weights at endpoint for each group (**P < 0.01 for SSG-H vs Control). (**D**) Body weight curves of mice in each group (*P < 0.05, **P < 0.01, ****P < 0.0001 for SSG groups vs Control). (**E**) H&E staining of tumor sections (representative images; 200X; high-dose SSG group shows fewer irregular tumor cells). (**F**) IHC staining of CD34 and Ki-67 in tumor tissues of each group, Scale bar, 50 μm, 200X (representative images; ***P < 0.001, ****P < 0.0001 for SSG vs Control). For Figure A, statistical analyses were conducted using ANOVA, while for the other figures, unpaired t-test was used (no multiple comparisons correction)

We first evaluated the effect of different SSG doses on tumor growth using the LLC xenograft mouse model. SSG treatment significantly suppressed tumor growth in a dose-dependent fashion, with higher doses of SSG producing more pronounced tumor inhibition (Fig. 2A–C). Throughout the 21-day treatment, SSG was well tolerated: all groups of mice showed stable body weights with no significant differences among the control and SSG-treated groups (Fig. 2D). Histological examination of tumor tissues revealed that higher doses of SSG led to a noticeable reduction in the proportion of abnormally shaped (irregular) tumor cells (Fig. 2E). Consistently, IHC staining showed that tumors from the high-dose SSG group had markedly lower expression of CD34 (vascular endothelial cell marker) and Ki-67 (proliferation markers) compared to tumors from control mice (Fig. 2F). These findings demonstrate that SSG exerts antitumor effects in vivo, and that a high dose of SSG is most effective at suppressing tumor growth and tumor cell proliferation. Accordingly, we selected the high-dose SSG regimen for subsequent experiments.

### SSG enhances the efficacy of anti-PD-1 therapy in tumor-bearing mice

**Fig. 3:**
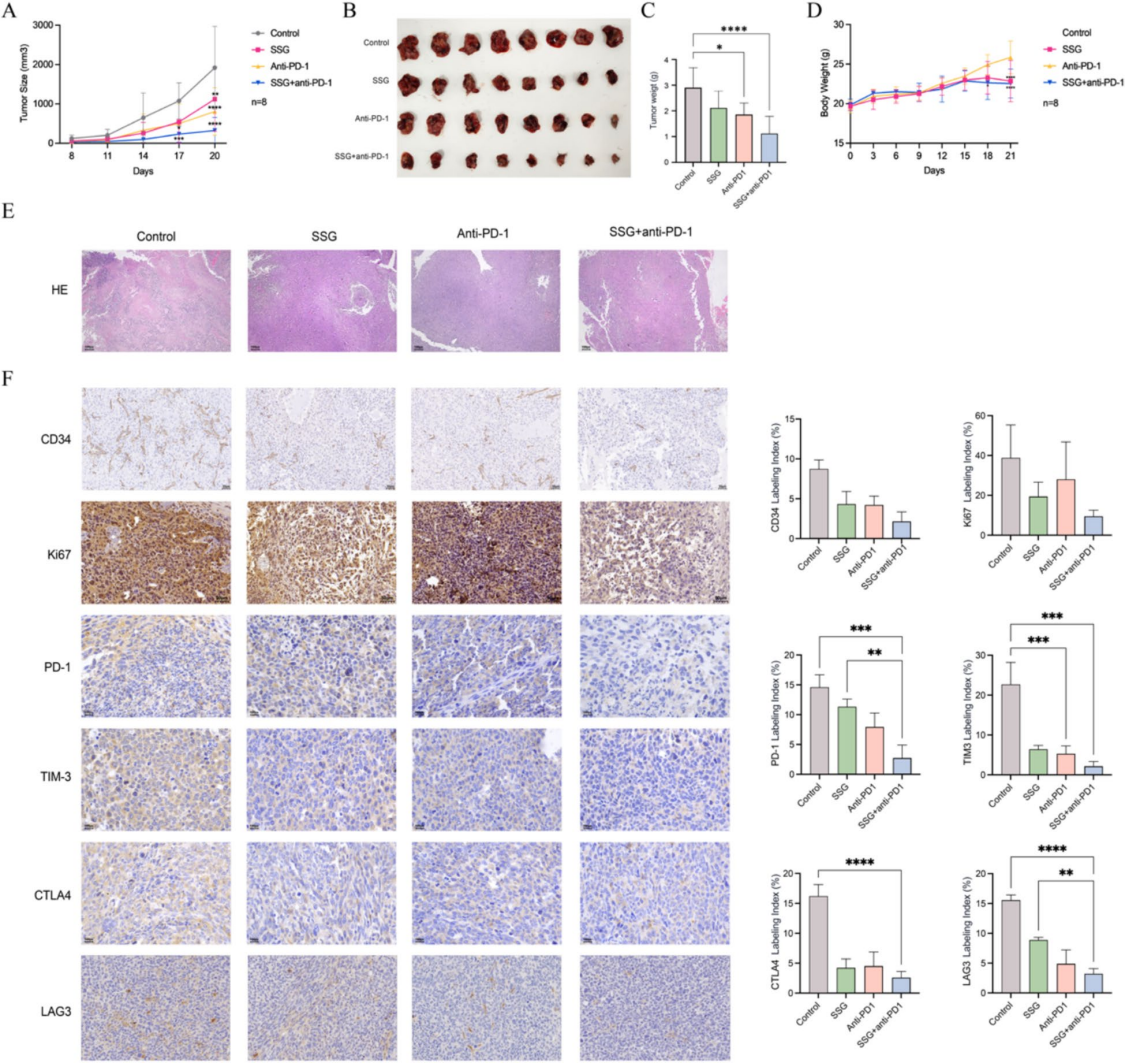
SSG boosts the anti-tumor efficacy of PD-1 blockade and reduces T cell exhaustion markers in vivo (n = 8). (A) Tumor volume curves for Control, SSG, anti-PD-1, and SSG + anti-PD-1 combination groups (*P < 0.05, **P < 0.01, ***P < 0.001, ****P < 0.0001 for combination vs Control at day 21). (B) Images of tumors excised from each group at the end of treatment. (C) Tumor weights at endpoint for each group (*P < 0.05, ****P < 0.0001 for combination vs Control). (D) Body weight changes over time (combination group shows a slight decrease relative to Control by day 21, *P < 0.05, ****P < 0.0001). (E) H&E staining of tumor tissues from each group (combo group shows more uniform tumor cell morphology), Scale bar, 100px. (F) IHC staining of CD34, Ki-67, and T cell exhaustion markers (PD-1, TIM-3, CTLA-4, LAG-3) in tumor tissues of each group, Scale bar, 50 μm, 200X (**P < 0.01, ***P < 0.001, ****P < 0.0001 for combination vs Control). For Figure A, statistical analyses were conducted using ANOVA, while for the other figures, unpaired t-test was used (no multiple comparisons correction)

We next examined whether SSG could augment the therapeutic efficacy of PD-1 blockade. In LLC tumor-bearing mice, SSG alone significantly inhibited tumor growth, and the combination of SSG with an anti-PD-1 antibody resulted in the greatest tumor suppression (Fig. 3**A**–**C**). By the end of the experiment, the combination treatment (SSG + anti-PD-1) achieved the smallest tumor volumes among all groups. During the treatment period, we monitored the body weights of the mice as an indicator of systemic effects. Mice receiving SSG or SSG + anti-PD-1 exhibited a slight divergence in weight gain starting around day 14, resulting in marginally lower body weights than control mice by day 21 (Fig. 3**D**). However, the weight differences were not accompanied by any overt signs of toxicity or distress.

Histopathology provided further evidence of enhanced efficacy in the combination group. H&E-stained tumor sections from the SSG + anti-PD-1 group showed more uniform, regular tumor cell morphology (cells were predominantly round or ovoid) compared to the more pleomorphic cells observed in controls (Fig. 3E). Moreover, IHC analysis demonstrated that the combination therapy greatly reduced the expression of proliferative markers in tumor tissues. CD34 and Ki-67 levels were dramatically lower in the SSG + anti-PD-1 group relative to both the control group and either monotherapy group (Fig. 3F). Importantly, the expression of T cell exhaustion markers in tumors was also lowest in the combination group: PD-1, TIM-3, CTLA-4, and LAG-3 staining in tumor-infiltrating lymphocytes was substantially reduced with SSG + anti-PD-1 treatment (Fig. 3F). These data indicate that SSG can potentiate anti-PD-1 immunotherapy, leading to stronger tumor growth inhibition and a more favorable tumor immune milieu than anti-PD-1 therapy alone.

### SSG enhances T cell infiltration and reduces T cell exhaustion in vivo

**Fig. 4:**
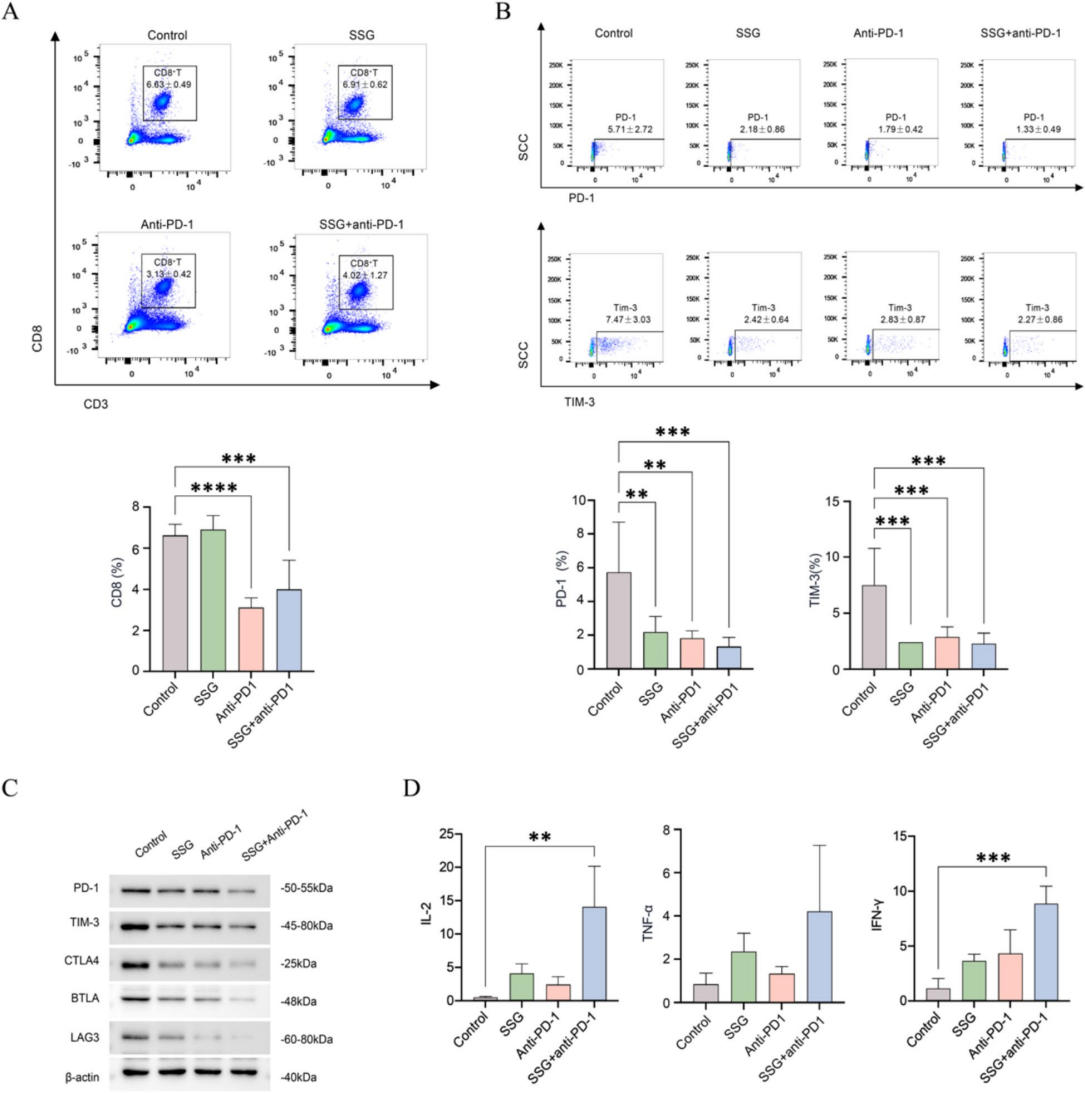
SSG reduces T cell exhaustion in vivo (n = 8). (**A**) Proportion of CD8^+^T cells in spleens (flow cytometry; ***P < 0.001, ****P < 0.0001 for SSG vs Control). (**B**) Mean fluorescence intensity (MFI) of PD-1 and TIM-3 on splenic CD8^+^T cells (**P < 0.01, ***P < 0.001 for combination vs monotherapy). (**C**) Western blot analysis of exhaustion-related proteins (PD-1, TIM-3, C TLA-4, BTLA, LAG-3) in tumor-infiltrating T cells (representative blots, showing lower levels in SSG and combination groups). (**D**) Levels of IL-2, TNF-α, and IFN-γ in serum or spleen homogenates of each group (**P < 0.01, ***P < 0.001 for SSG and combo vs Control). Statistical analyses were conducted using unpaired t test (no multiple comparisons correction)

To assess SSG’s impact on T cells in the context of PD-1 therapy, we analyzed immune cell populations and cytokines in treated mice. Flow cytometry of spleen samples showed that anti-PD-1 monotherapy slightly increased certain immune responses but was paradoxically associated with a reduction in the proportion of splenic CD8^+^T cells (possibly reflecting T cell redistribution or negative feedback). In contrast, mice treated with SSG had a significantly higher percentage of CD8^+^ T cells in the spleen, and the combination of SSG + anti-PD-1 adjusted the frequency of CD8⁺T cells to a level closer to that of the control group (though slightly lower than the control in this cohort) (Fig. 4A). We next examined T cell “exhaustion” status by measuring key inhibitory receptors on CD8^+^ T cells. SSG treatment led to lower expression of exhaustion markers, and the addition of SSG to anti-PD-1 therapy had an additive effect. In the combination group, surface levels of PD-1 and TIM-3 on CD8^+^T cells were markedly reduced compared to either SSG or anti-PD-1 alone (Fig. 4B). Similarly, the combination group showed the lowest expression of other inhibitory receptors (e.g., CTLA-4, BTLA, LAG-3) on T cells (Fig. 4C). Consistent with the improved T cell profiles, SSG-treated mice (especially when combined with PD-1 blockade) had significantly elevated levels of Th1-type cytokines. ELISA results from serum (or spleen homogenates) demonstrated that IL-2, TNF-α, and IFN-γ concentrations were higher in the SSG and SSG + anti-PD-1 groups than in controls, with the combination therapy yielding the highest cytokine levels (Fig. 4D). These findings suggest that SSG enhances anti-tumor immunity in vivo by expanding the CD8^+^T cell pool, relieving T cell exhaustion, and promoting a pro-inflammatory cytokine environment.

**Fig. 5:**
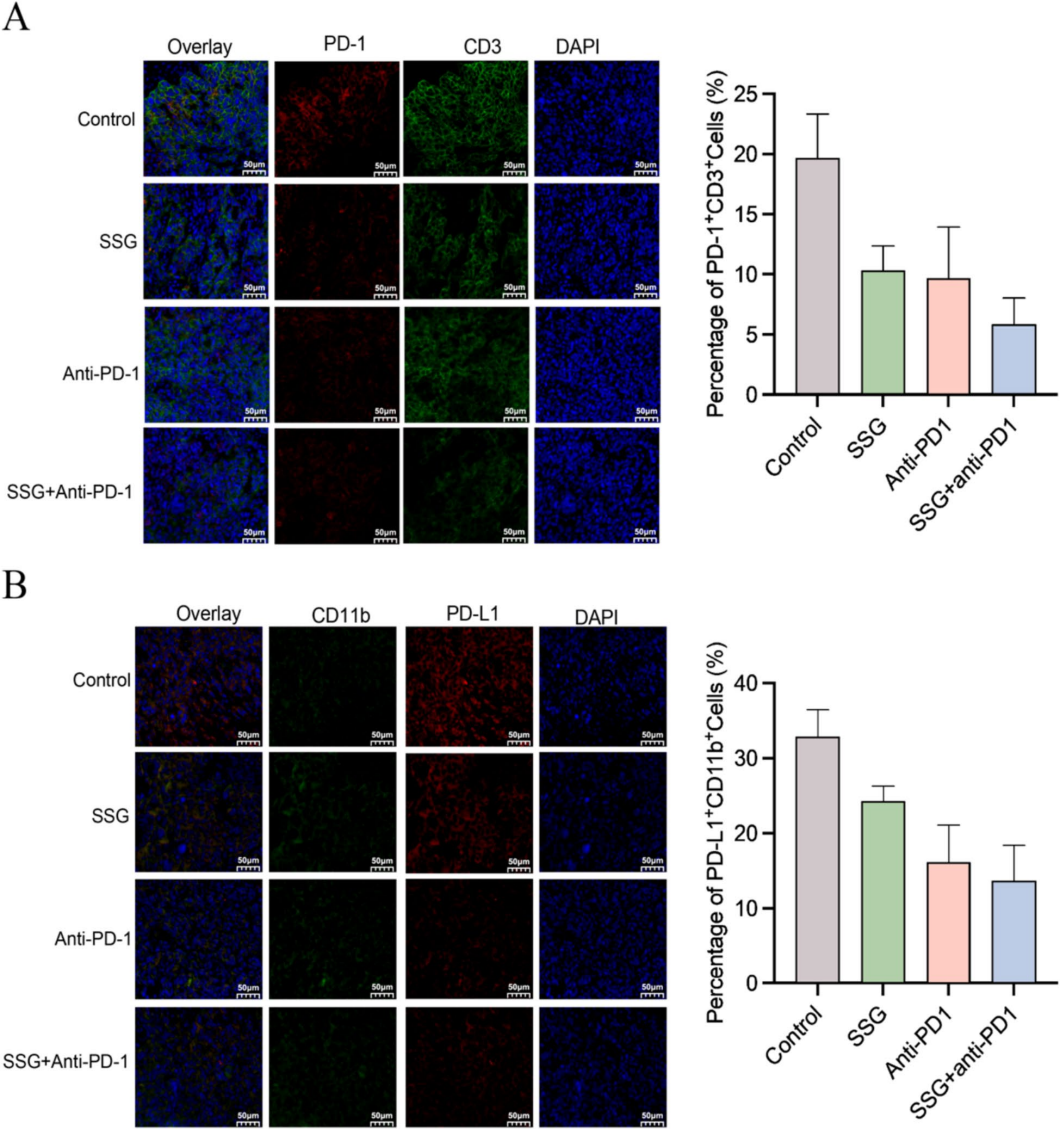
Immunofluorescence analysis of PD-1/PD-L1 in tumor tissues (n = 8). (**A**) Representative confocal images of PD-1 (green) and CD3 (red) co-staining in tumors from each group (nuclei in blue). In the Control group, many CD3^+^T cells co-express PD-1 (yellow overlay), whereas in the combination group, far fewer PD-1^+^T cells are seen. Scale bar, 50 μm, 200X. (**B**) Representative images of PD-L1 (green) and CD11b (red) staining. PD-L1 is present in tumor tissues but shows minimal overlap with CD11b^+^myeloid cells, and overall PD-L1 signal is reduced in the SSG and combination groups, Scale bar, 50 μm, 200X

Double immunofluorescence staining of tumor sections provided visual confirmation of these immune changes. In control tumors, there was strong co-localization of PD-1 with CD3^+^T cells, indicating that many tumor-infiltrating T cells were PD-1^high^ (exhausted). In contrast, SSG-treated tumors (especially those also treated with PD-1 antibody) showed markedly fewer PD-1^+^T cells (reduced PD-1/CD3 co-localization). We did not observe significant co-localization of PD-L1 with CD11b in control tumors, suggesting that in this model, PD-L1 may be expressed more on tumor or other cells than on MDSCs. Notably, SSG treatment (± anti-PD-1) appeared to reduce overall PD-L1 expression in the tumor tissue, with the most pronounced reduction seen in the combination group (as qualitatively shown in Fig. 5). These microscopy results align with the flow cytometry and IHC data, indicating that SSG contributes to an immune-permissive tumor environment by modulating both T cells and suppressive myeloid cells.

### SSG reduces MDSC accumulation and suppresses MDSC immunosuppressive functions in vivo

**Fig. 6:**
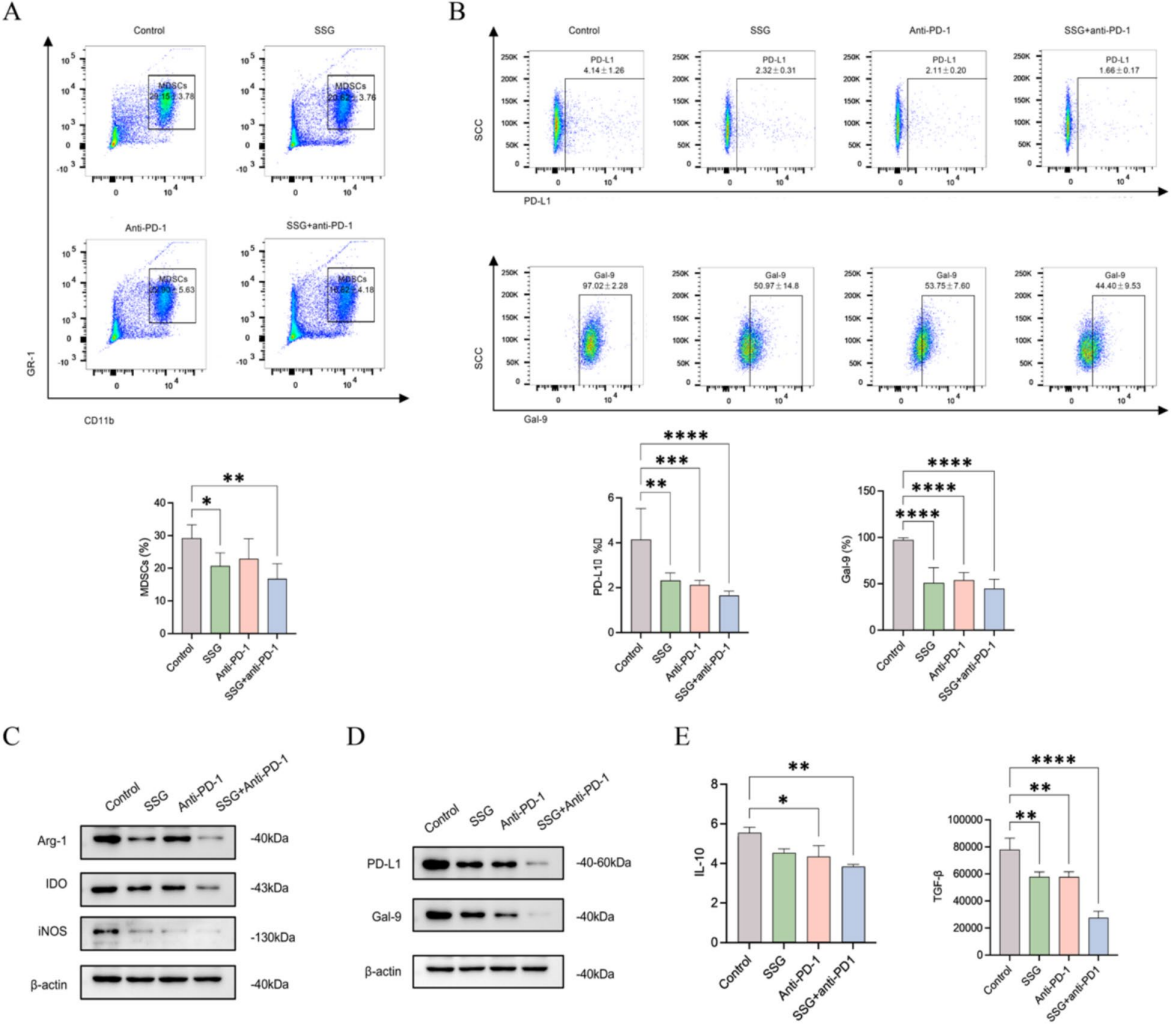
SSG inhibits MDSC accumulation and function in vivo (n = 8). (**A**) Frequency of MDSCs (CD11b^+^Gr-1^+^cells) in spleens of each group, measured by flow cytometry (*P < 0.05, **P < 0.01 vs Control). (**B**) Expression of PD-L1 and Gal-9 on splenic MDSCs (MFI values); **P < 0.01, ***P < 0.001, ****P < 0.0001, showing significant reductions in combination group. (**C**) Western blot of MDSC-related enzymes (Arg-1, IDO, iNOS) in tumor tissue; SSG, especially with anti-PD-1, greatly reduces these proteins. (**D**) Western blot of exhaustion ligands (PD-L1, Gal-9) in tumor tissues of each group, showing lowest levels in SSG + anti-PD-1 group. (**E**) IL-10 and TGF-β concentrations in serum (or tumor microenvironment) of each group, measured by ELISA (*P < 0.05, **P < 0.01, ****P < 0.0001). Statistical analyses were conducted using unpaired t test (no multiple comparisons correction)

We then investigated how SSG affects MDSCs in tumor-bearing mice. Flow cytometric analysis of splenocytes revealed that mice treated with SSG had significantly fewer MDSCs (identified as CD11b^+^Gr-1^+^cells) compared to control mice (Fig. 6A). In control mice, splenic MDSCs expressed high levels of PD-L1 and Galectin-9 on their surface. Treatment with SSG led to a notable downregulation of these exhaustion-inducing ligands on MDSCs, and the combination of SSG with anti-PD-1 resulted in the most significant reductions in PD-L1 and Gal-9 levels (Fig. 6B). These data suggest that SSG not only reduces the number of MDSCs but also impairs the ability of any remaining MDSCs to inhibit T cells. Key MDSC-associated enzymes that mediate immune suppression—Arg-1, IDO, and iNOS—were all markedly downregulated in the SSG-treated groups, with the greatest decrease observed in the combination therapy group (Fig. 6C). In addition, the combination group showed substantially lower levels of PD-L1 and Gal-9 proteins in tumor tissue (reflecting contributions from both MDSCs and tumor cells) compared to controls (Fig. 6D). SSG treatment alone also reduced PD-L1 and Gal-9 levels relative to control. Collectively, these results indicate that SSG administration (either alone or in combination) effectively reduces the overall levels of immunosuppressive mediators in the tumor microenvironment, contributing to the alleviation of the immunosuppressive state. Functionally, we assessed whether SSG’s effects on MDSCs translated into changes in cytokine production. ELISA measurements indicated that MDSCs from SSG-treated mice secreted significantly lower amounts of IL-10 and TGF-β—two potent immunosuppressive cytokines—compared to MDSCs from control mice (Fig. 6E). Again, the combination of SSG with anti-PD-1 had the strongest effect, virtually normalizing the levels of these cytokines to baseline. Collectively, these results demonstrate that SSG can attenuate both the abundance and the suppressive activity of MDSCs in vivo, which likely contributes to the improved T cell responses and anti-tumor effects observed with SSG treatment.

### Chemical components identified in SSG-containing serum

**Fig. 7:**
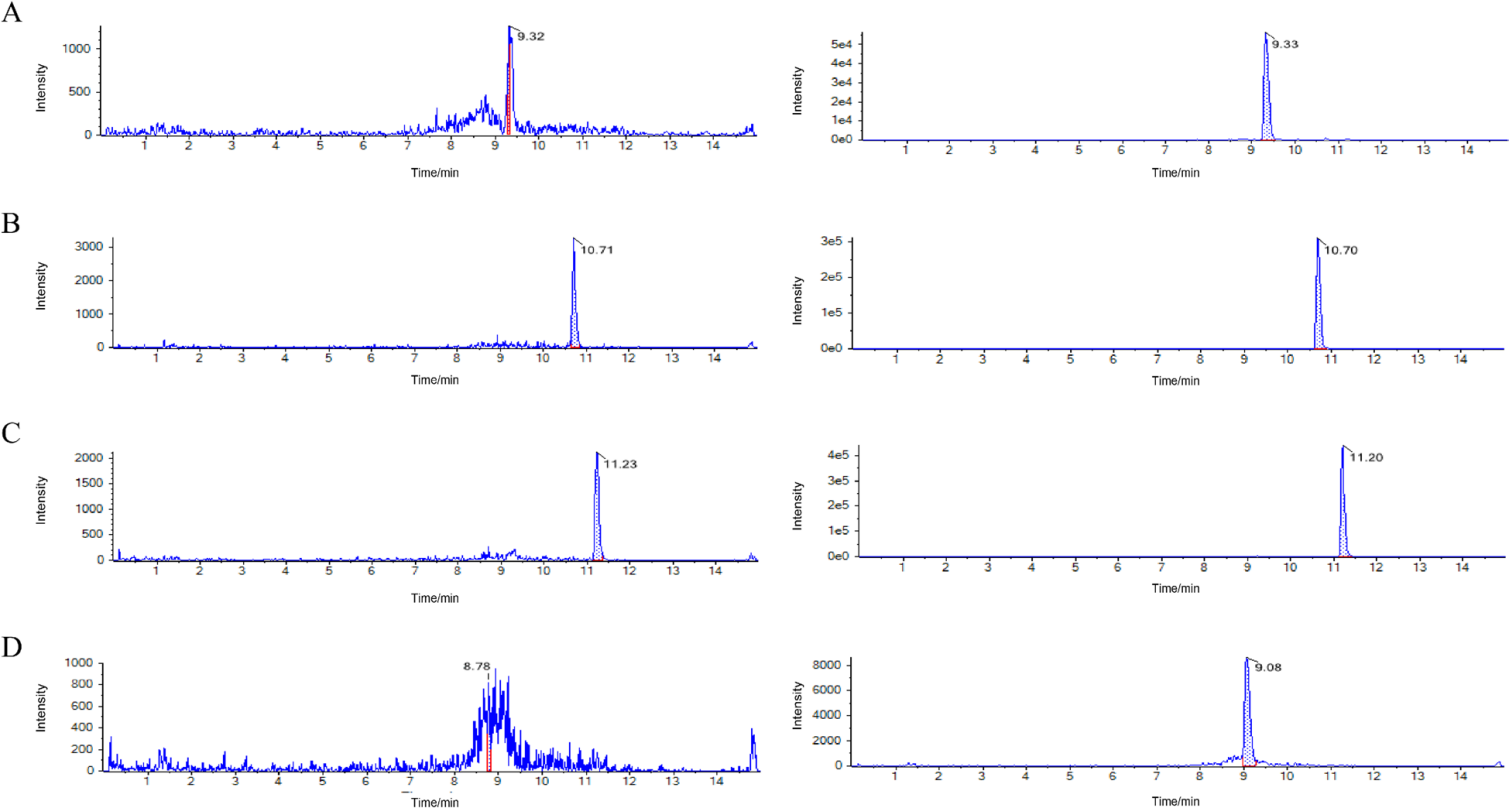
MRM chromatograms of blank serum vs. SSG-containing serum. (**A**) Chromatogram for ginsenoside Rg1; (**B**) Rb1; (**C**) Rd; (**D**) notoginsenoside R1. These profiles confirm the presence of SSG’s major ginsenosides in the serum of treated rats (peaks indicated by arrows)

To facilitate the in vitro assays, we prepared SSG-containing serum from rats and confirmed the presence of key active compounds in the serum. Using UPLC-MS/MS in line with Chinese Pharmacopoeia guidelines (2015 edition), we verified that the prepared serum contained the expected ginsenosides within pharmacopoeial standards. To ensure the reliability of the serum component analysis, in this study, the Total Ion Chromatogram (TIC) of serum containing SSG was obtained by HPLC–MS/MS (Fig. S1). This chromatogram showed that all components were effectively separated within the retention time range of 1.0–12.0 min. Based on this, HPLC–MS/MS analysis identified the primary constituents of the SSG-containing serum, and representative MRM chromatograms for each compound are shown in Fig. 7. Standard curves (Table 4) were generated for quantification, and the concentrations of major ginsenosides in the serum were determined by correlating chromatographic peak areas with these standard curves. As summarized in Table 5, the serum from SSG-treated rats contained approximately 12.92 ng/mL of ginsenoside Rg1, 1723.06 ng/mL of ginsenoside Rb1, 412.56 ng/mL of ginsenoside Rd, and 13.37 ng/mL of notoginsenoside R1. These data confirm that oral administration of SSG leads to systemic exposure of its active ingredients (at least in metabolite form), which provides a rationale for our in vitro experiments using drug-containing serum. While using serum from SSG-treated animals makes the in vitro findings more physiologically relevant, it should be noted that detailed pharmacokinetic studies were not conducted here. In future studies, a more comprehensive pharmacokinetic profiling of SSG’s constituents would be valuable to correlate specific component levels with biological effects, thereby guiding dose optimization and translational prospects.

**Table 4:** Results of standard curve determination of main components in SSG-containing serum

Component	Linear regression equation	Correlation coefficient		Linear range ng/mL
Ginsenoside Rg1	Y = 4.65 × 10^4^X-2.85 × 10^5^		0.9968	4.88 ~ 156.25
Ginsenoside Rb1	y = 1.21 × 10^3^X-1.31 × 10^5^		0.9970	9.76 ~ 5000
Ginsenoside Rd	y = 7.79 × 10^3^X-8.24 × 10^5^		0.9918	9.76 ~ 5000
Notoginsenoside R1	y = 1.155 × 10^4^X-8.42 × 10^4^		0.9964	4.88 ~ 156.25

**Table 5:** Concentrations of main components in SSG-containing serum

Component	Concentration (ng/mL)
Ginsenoside Rg1	12.9237
Ginsenoside Rb1	1723.0593
Ginsenoside Rd	412.5584
Notoginsenoside R1	13.3738

### SSG-containing serum suppresses MDSC immunosuppressive activity and reverses T cell exhaustion in vitro

**Fig. 8:**
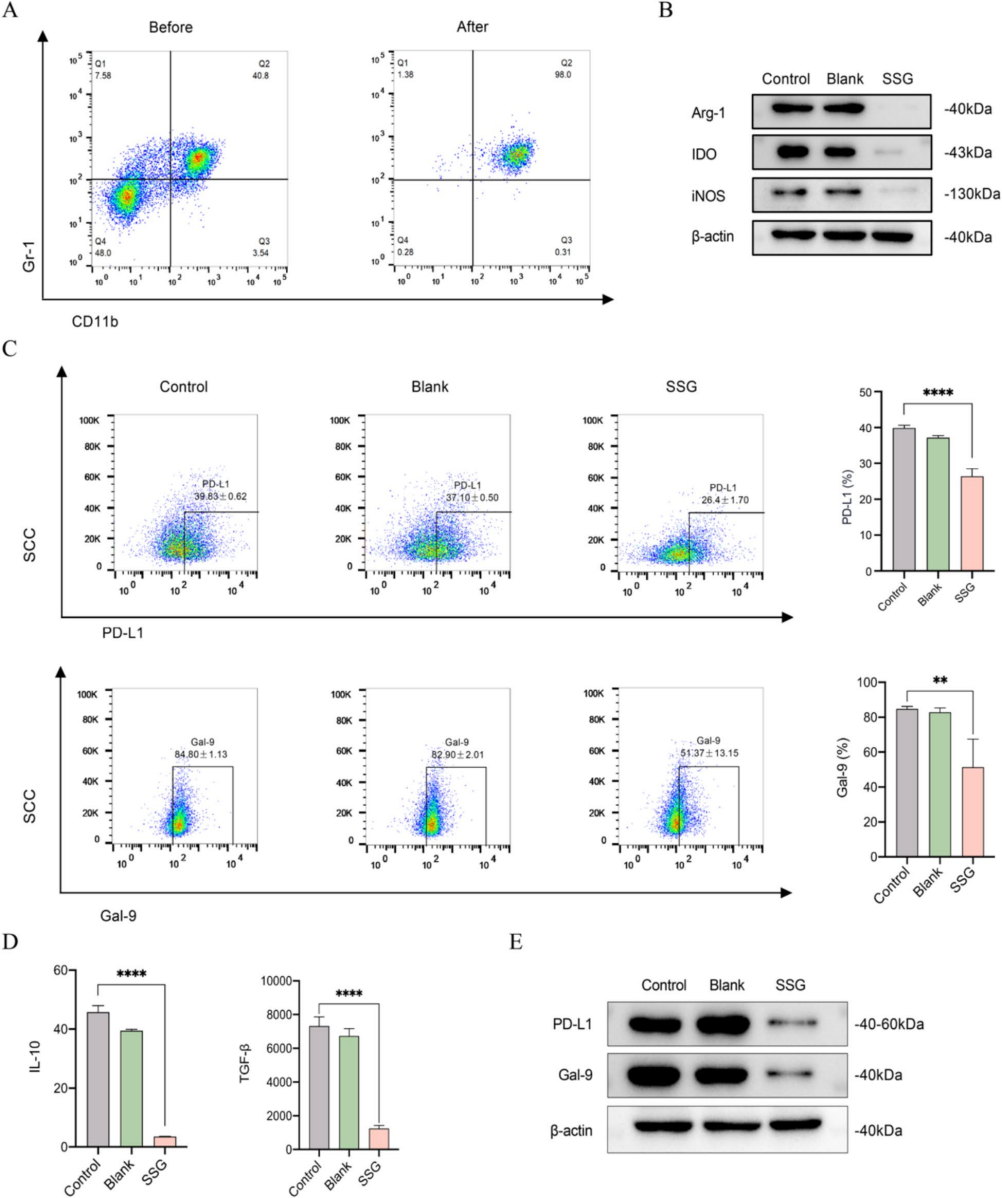
SSG-containing serum reduces the immunosuppressive activity of MDSCs in vitro (n = 8). (**A**) Flow cytometry dot plots showing the percentage of MDSCs (CD11b^+^Gr-1^+^) before and after expansion, confirming > 95% purity. (**B**) Quantified expression of Arg-1, IDO, and iNOS in MDSCs (by Western blot or flow cytometry) with and without SSG serum treatment; SSG significantly decreases all three. (**C**) Surface levels of PD-L1 and Gal-9 on MDSCs after treatment (flow cytometry MFI), **P < 0.01, ****P < 0.0001 vs untreated MDSCs. (**D**) Secretion of IL-10 and TGF-β by MDSCs, measured by ELISA; ****P < 0.0001. (**E**) Western blot confirming reduced PD-L1 and Gal-9 protein in MDSCs treated with SSG serum (representative blots). Statistical analyses were conducted using unpaired t test (no multiple comparisons correction)

We modeled the interaction between MDSCs and T cells in vitro to further dissect the mechanism of SSG. MDSCs were derived from mouse bone marrow in culture (yielding a > 95% pure CD11b^+^Gr-1^+^ population, Fig. 8A) and then treated with SSG-containing serum. This treatment significantly diminished the immunosuppressive features of the MDSCs. Specifically, after exposure to SSG-conditioned serum, MDSCs showed markedly lower expression of Arg-1, IDO, and iNOS compared to untreated MDSCs (Fig. 8B). Flow cytometry also revealed that the surface expression of PD-L1 and Gal-9 on MDSCs was greatly reduced following SSG serum treatment (Fig. 8C). Correspondingly, the amounts of IL-10 and TGF-β released by MDSCs into the culture medium were significantly decreased (as measured by ELISA, Fig. 8D). These results are in line with our in vivo observations, indicating that SSG can directly impair MDSC-derived suppressive factors. Western blotting of MDSC lysates provided further confirmation: protein levels of PD-L1 and Gal-9 were lower in MDSCs treated with SSG-containing serum than in control MDSCs (Fig. 8E).

**Fig. 9:**
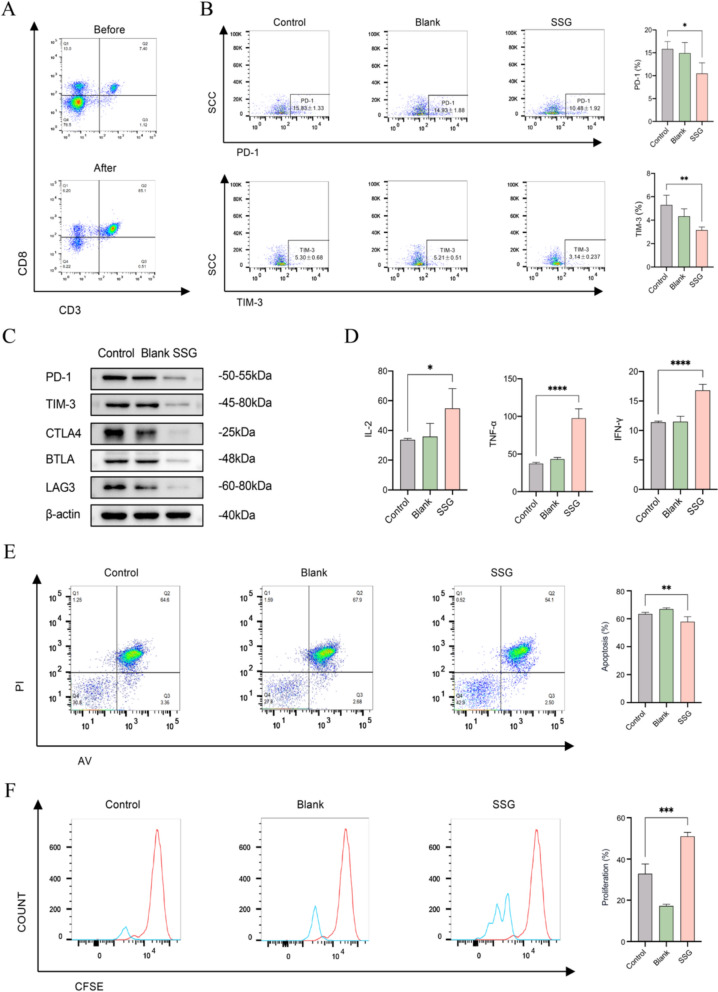
SSG-treated MDSCs alleviate T cell exhaustion in co-culture. (**A**) Flow cytometry sorting of CD8^+^T cells from mouse spleens (pre-sort and post-sort purity plots). (**B**) Expression of PD-1 and TIM-3 on CD8^+^T cells after co-culture with MDSCs ± SSG treatment (*P < 0.05, **P < 0.01 vs co-culture with untreated MDSCs). (**C**) Western blot analysis of CTLA-4, BTLA, LAG-3 in CD8^+^T cells after co-culture, showing lower levels when co-cultured with SSG-treated MDSCs. (**D**) Levels of IL-2, IFN-γ, and TNF-α in co-culture supernatants, reflecting enhanced cytokine production in the presence of SSG-treated MDSCs (*P < 0.05, ****P < 0.0001). (**E**) Apoptosis rates of CD8^+^T cells after 24 h co-culture, with significantly fewer Annexin V^+^cells when MDSCs were pretreated with SSG (**P < 0.01). (**F**) Proliferation of CD8^+^T cells (CFSE dilution after 72 h co-culture), showing increased proliferation index with SSG-treated MDSCs (***P < 0.001). Statistical analyses were conducted using unpaired t test (no multiple comparisons correction)

Next, we investigated whether these changes in MDSCs could alleviate T cell exhaustion. Purified CD8 + T cells were co-cultured with MDSCs in a Transwell system. In co-cultures where MDSCs had been pretreated with SSG-containing serum, we observed a clear improvement in T cell indicators compared to co-cultures with untreated MDSCs (Fig. 9A). Flow cytometry showed that CD8^+^T cells co-cultured with SSG-treated MDSCs had significantly lower levels of PD-1 and TIM-3 on their surface (Fig. 9B), suggesting reduced exhaustion. Consistently, Western blot analysis of these CD8^+^T cells indicated reduced expression of other exhaustion markers (CTLA-4, BTLA, LAG-3) when the T cells were influenced by SSG-treated MDSCs (Fig. 9C). Functionally, T cells in the presence of SSG-treated MDSCs were more active: the production of key anti-tumor cytokines (IL-2, IFN-γ, TNF-α) was higher in these co-cultures **(**Fig. 9D). Moreover, the CD8^+^T cells co-cultured with treated MDSCs showed lower apoptosis rates and higher proliferation (as evidenced by Annexin V/PI staining and CFSE dilution, respectively) compared to T cells co-cultured with control MDSCs (Fig. 9E, F). Taken together, these in vitro findings demonstrate that SSG (via factors present in drug-containing serum) can relieve MDSC-induced T cell suppression—by both disabling MDSC suppressive mechanisms and protecting T cells from exhaustion—thereby restoring T cell proliferative capacity and survival.

### Potential therapeutic targets of SSG for LUAD

**Fig. 10:**
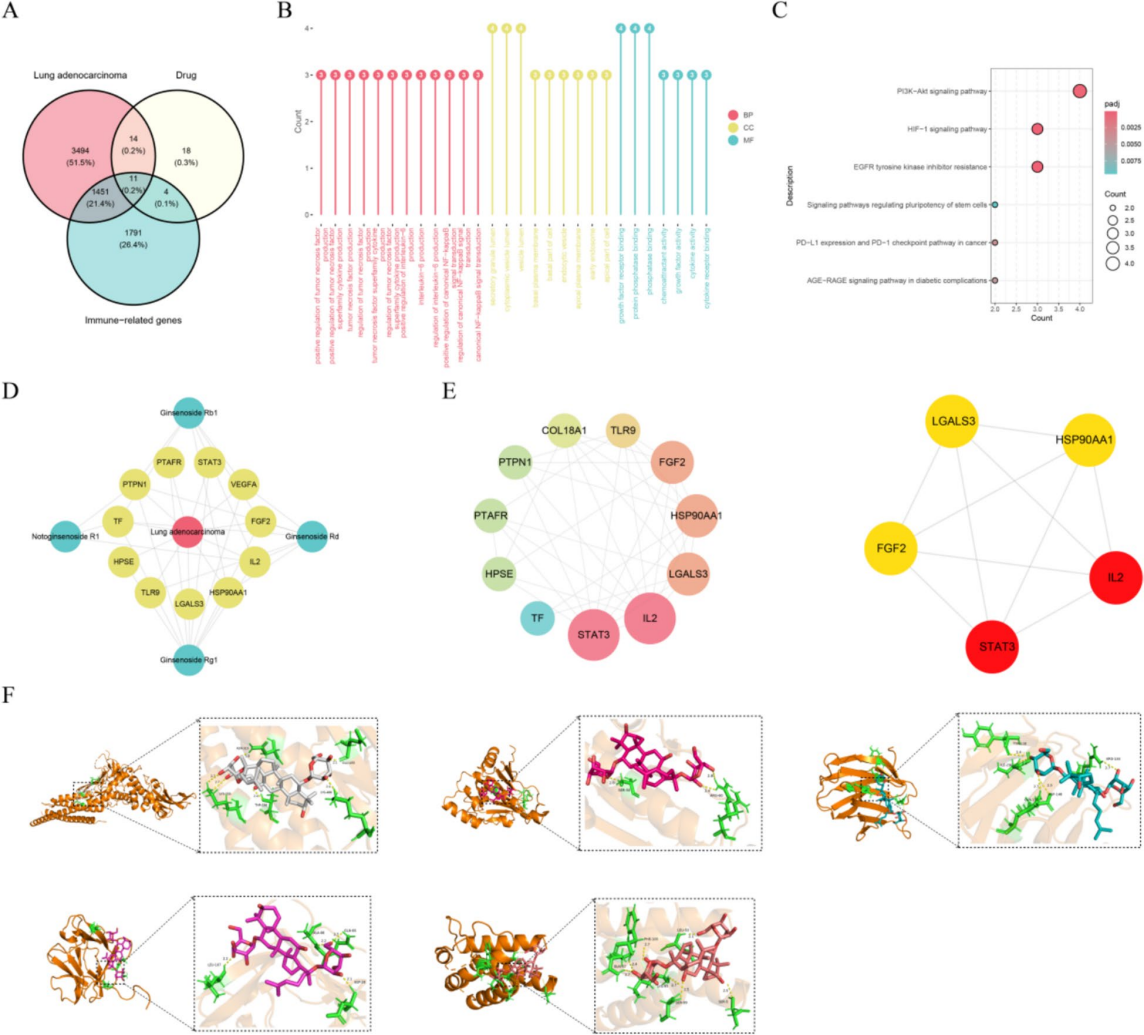
Network pharmacology analysis of SSG’s immune-related mechanisms. (**A**) Overlap between SSG compound targets, lung adenocarcinoma (LUAD) targets, and immune-related genes (Venn diagram). (**B**) Herb–component–target–disease interaction network for SSG. (**C**) Protein–protein interaction (PPI) network of common targets (minimum confidence score 0.7). (**D**) Top 20 KEGG pathways enriched by SSG targets. (**E**) GO enrichment results for SSG targets (biological process, molecular function, cellular component). Statistical analyses were conducted using Hypergeometric test, and the P-values were adjusted for multiple comparisons using the Benjamini-Hochberg (BH) method. (**F**) Molecular docking of key SSG compounds with core targets (illustration of one compound–target pair)

Ginsenoside Rg1, Ginsenoside Rb1, Ginsenoside Rd, and Notoginsenoside R1 were selected for network pharmacology analysis. After selecting of 47 targets for the active ingredients, we identified 11 common targets that overlap among 47 active compounds’ targets, 3,257 immune-related genes, and 4,970 LUAD–related genes (Fig. 10A). GO enrichment analysis revealed these common targets were associated with the regulation of inflammatory immune signals, cellular secretion and substance transport, as well as the binding and activity mediation of signal molecules (Fig. 10B). As for KEGG pathways, they were mainly involved in functions such as cell signal transduction (such as PI3K-Akt, HIF-1 pathway), tumor drug resistance (such as EGFR tyrosine kinase inhibitor resistance), and immune checkpoint regulation (such as PD-L1 expression and PD-1 checkpoint pathway in tumors) (Fig. 10C). Among them, the number (Count) and enrichment significance (padj) of enriched genes in pathways such as PI3K-Akt and HIF-1 were relatively higher.

To more intuitively illustrate the targeting relationships among common targets, active ingredients, and LUAD, a compound–target–disease network was constructed to visualize these interactions (Fig. 10D), where nodes represent herbs, chemical components, targets, or diseases, and edges indicate their relationships. We next adopted a low confidence threshold of 0.15 to characterize the interactions among common targets, and ranked the genes via the degree algorithm to select the top 5 hub targets, including IL2, STAT3, HSP90AA1, LGALS3, and FGF2, which may play central roles in SSG’s pharmacological effects (Fig. 10E).

Molecular docking simulations provided additional support for these network findings. Ginsenoside Rg1 was docked with 5 hub targets. The docking results showed favorable binding interactions; for instance, Ginsenoside Rg1 was predicted to bind strongly to LGALS3 (estimated binding free energy = –7.0 kcal/mol) (Fig. 10F, Table 6).

**Table 6:** Molecular docking of Ginsenoside Rg1 with 5 hub targets

UniProt	Target	Compound	Energy	Hydrogen bond
8SOZ	IL2	Ginsenoside Rg1 (CID: 441,923)	-6.1	7
6NJS	STAT3		-6.8	6
8X2R	HSP90AA1		-5.8	3
8ITZ	LGALS3		-7.0	5
8OM6	FGF2		-6.3	4

### Validation of Hub Gene Expression and PI3K-Akt Signaling Pathway

**Fig. 11:**
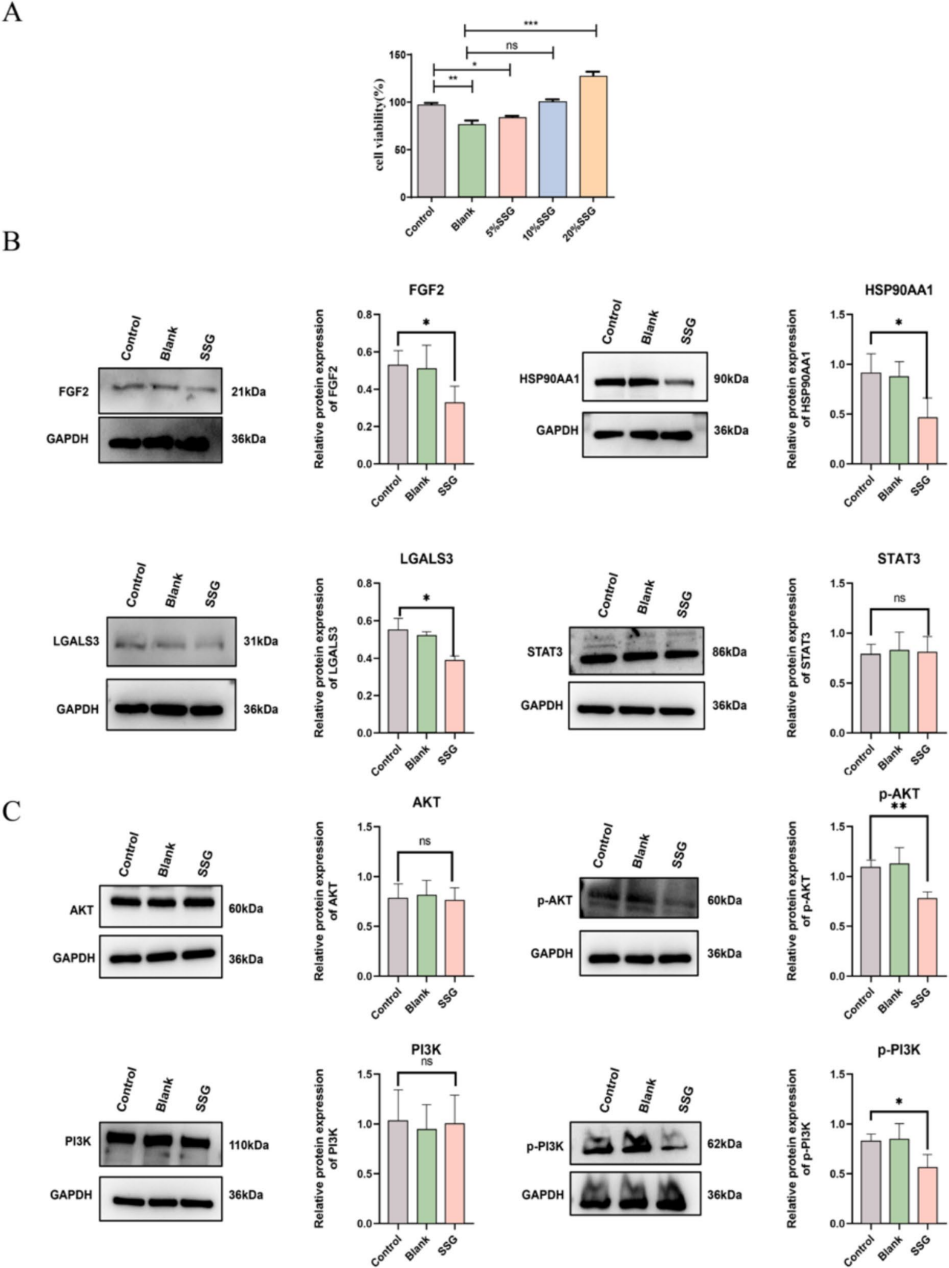
Validation of hub targets and PI3K-Akt pathway proteins by Western blot. (**A**) Cell viability of MDSCs in serum containing different concentrations of drugs (ns means not significant, *P < 0.05, **P < 0.01, ***P < 0.001). (**B**) Western blot results of hub targets (ns means not significant, *P < 0.05). (**C**) Western blot results of PI3K-Akt pathway proteins (ns means not significant, *P < 0.05, **P < 0.01). Statistical analyses were conducted using unpaired t test (no multiple comparisons correction)

To explore the potential pathways of SSG in the treatment of LUAD in this study, MDSCs were intervened with different concentrations of SSG-containing serum for 24 h, and the cell viability was detected by the CCK-8 method. The results showed that the cell viability of the 20% SSG drug-containing serum group reached its peak, and thus was used for subsequent experiments for verification (Fig. 11A). Subsequently, the protein expression level of the hub target in MDSCs was detected by Western blot. The results indicated that the expression of the internal reference protein was normal, but the IL2 protein was not detected. Compared with the control group, the protein expression levels of HSP90AA1, LGALS3 and FGF2 in the drug-containing serum treatment group were significantly decreased (Fig. 11B).

Given that the common targets of SSG active ingredients, immune-related genes and LUAD-related genes were significantly enriched in the PI3K-Akt pathway, we further utilized Western blot to detect the expression differences of proteins related to this pathway in the SSG treatment group. The results showed that compared with the control group, the protein expression levels of p-PI3K and p-Akt in the drug-containing serum treatment group were significantly down-regulated (Fig. 11C). These results further suggested that the PI3K-Akt pathway might mediate the regulatory effect of SSG.

## Discussion

Combining TCM formulations with immune checkpoint inhibitors (ICIs) has shown synergistic potential in multiple cancer models. SSG containing Panax quinquefolius (American ginseng), Panax notoginseng, and Cordyceps sinensis, has been clinically used as an adjuvant to improve treatment tolerance. Ginseng saponins and cordycepin are known to enhance immunity and exhibit anti-tumor effects [24, 25]. We previously reported that SSG inhibited lung cancer metastasis by interfering with MDSC differentiation [18]. The present study extends these observations by demonstrating that SSG not only reduces markers of T cell exhaustion but also improves the efficacy of PD-1 blockade, effectively reshaping the tumor microenvironment toward immune-mediated control. Notably, mice receiving high-dose SSG showed a trend of body weight loss toward the end of treatment. Although modest and not accompanied by overt toxicity, this observation suggests that prolonged or high-dose exposure may induce mild metabolic alterations–underscoring the importance of dose optimization. Defining an optimal therapeutic window (efficacy retained with minimal adverse effects) is critical for SSG’s clinical translation, and future studies should evaluate whether reduced dosing or shorter regimens preserve efficacy while mitigating side effects. Clinically, dose-escalation trials and close monitoring of body weight and general health will be essential when combining SSG with ICIs. As a complex Chinese medicinal formula, SSG comprises multiple bioactive constituents. Thus, we cannot attribute the observed immunomodulatory and anti-tumor effects to any single component. Instead, these effects likely arise from synergistic or additive interactions among various ginsenosides and active ingredients, which collectively modulate the tumor immune microenvironment rather than acting via a single dominant mechanism.

Our results further underscore the pivotal role of MDSCs in immunotherapy resistance. Elevated MDSC frequencies have been associated with poor responses to PD-1/PD-L1 inhibitors in NSCLC patients [26]. These cells foster a suppressive milieu by limiting T cell activation and promoting additional immunosuppressive subsets [27]. We focused on MDSC-derived mediators—Arg-1, IDO, iNOS, PD-L1, and Galectin-9—because they represent major effector arms of suppression. Arg-1 and IDO deplete amino acids required for T cell proliferation [28, 29], while iNOS produces nitric oxide that impairs T cell function [30]. In parallel, PD-L1 and Gal-9 interact with inhibitory receptors such as PD-1 and TIM-3, reinforcing T cell exhaustion [31]. MDSCs secrete factors such as TGF-β, IL-10, and IDO, which boost the immunosuppressive activity of effector T cells, facilitate immune evasion, and contribute to T cell exhaustion [32–35]. Our findings that SSG reduced these mediators indicate that it can disrupt multiple layers of MDSC-driven suppression.

Importantly, SSG treatment not only diminished immunosuppressive factors but also enhanced cytokine production by CD8⁺T cells (IL-2, IFN-γ, TNF-α). CD8⁺ T cells can kill LUAD cells and regulate tumor growth, and their infiltration level is positively correlated with the efficacy of anti-PD-1/PD-L1 immunotherapy [36]. Similarly, Hirsutella sinensis fungus (HSF), an artificial substitute for Cordyceps sinensis, a core component of SSG, promotes M1 polarization of macrophages via upregulating CCRL2 expression, thereby activating the antitumor activity of CD8⁺ T cells and ultimately suppressing lung cancer progression [17]. Notably, Sanqi Oral Liquid, a herbal formulation that also contains Panax notoginseng but differs from SSG in terms of component compatibility and administration dosage, has been demonstrated to reduce CD8⁺ T cell expression levels [37]. When we assessed the exhaustion status of CD8⁺ T cells by detecting the key inhibitory receptors on their surface, we found that SSG treatment reduced the expression of exhaustion markers, while the combination of SSG and anti-PD-1 therapy exerted a synergistic effect, leading to even lower expression levels of PD-1 and TIM-3. The changes of T cell exhaustion markers (PD-1, TIM-3) suggest that the function of T cells may have recovered [38]. Taken together, these results highlight the potential of SSG as an adjuvant therapy for lung adenocarcinoma, as it can optimize CD8⁺ T cell function and synergize with immune checkpoint inhibitors, while also underscoring the importance of rational formulation design for herbal medicines in cancer immunotherapy.

To explore the potential molecular pathways underlying SSG-mediated effects on LUAD, we predicted its core functional targets and subsequently performed functional enrichment analyses. Results from GO and KEGG enrichment assays indicated that these common targets were significantly enriched in the PI3K-Akt signaling pathway, suggesting that this pathway may play a pivotal role in SSG-regulated LUAD progression. The PI3K/Akt signaling pathway plays a pivotal role in tumorigenesis by regulating cell survival, proliferation, migration and apoptosis, and its aberrant activation drives tumor initiation and metastasis [39]. In our study, the protein expression levels of p-PI3K and p-Akt in the SSG group were significantly lower than those in the control group, suggesting that SSG may exert a therapeutic effect on LUAD by inhibiting the PI3K/Akt signaling pathway. The PI3K-Akt pathway plays a crucial role in the functions, migration and metabolism of MDSCs [40]. Previous studies have shown that in the treatment of melanoma, artemisinins (ARTs) modulate the functional polarization of MDSCs by activating the p70 S6K/mTOR signaling pathway while inhibiting the PI3K/Akt and MAPK pathways [41]. In murine models, ART administration not only suppresses MDSC activity but also enhances the infiltration of CD3⁺ and CD8⁺ T cells into the tumor microenvironment, thereby exerting a potent anti-tumor effect to reduce tumor growth [41]. Furthermore, the evidence indicates that inhibition of PI3Kδ promotes the expansion of the TCF-1^+^ progenitor-like exhausted T-cell (Tpex) subpopulation [42]. Collectively, these results suggest that the PI3K/AKT signaling pathway may serve as a potential mechanism contributing to the anti-LUAD effects of SSG. Given the critical roles of the PI3K/AKT pathway in regulating MDSC function, CD8^+^ T cell infiltration, and the generation of TCF-1^+^ progenitor-like exhausted T cells, we hypothesize that SSG may also elicit anti-tumor immunity by modulating these immune components.

Finally, the translational implications of our findings are significant. MDSCs are increasingly recognized as predictive biomarkers for immunotherapy outcomes in NSCLC, with elevated frequencies correlating with poor responses to PD-1/PD-L1 blockade [22, 31]. By demonstrating that SSG reduces both MDSC abundance and their suppressive mediators, our study suggests that SSG may not only enhance ICI efficacy but also support the use of MDSC levels as a predictive biomarker to guide patient stratification. This dual therapeutic and diagnostic potential reinforces the rationale for integrating herbal adjuvants such as SSG with immune checkpoint therapy in lung cancer.

The current research has obvious limitations in terms of safety assessment.

The lack of pathological examination and serum biochemical analysis of key organs means that the underlying causes of mild weight loss in the high-dose SSG group*–*especially its potential association with treatment-related toxicity–remain to be clarified. Further systematic investigation is needed, including a comprehensive security assessment, to address this knowledge gap. This study conducted experiments using in vitro induced-differentiated bone marrow-derived MDSCs. However, the effect of SSG intervention on the regulation of tumor-infiltrating MDSCs on the function of CD8⁺ T cells has not been directly explored. The functional and causal link between SSG-induced PI3K-Akt pathway inhibition (confirmed by Western blot) and its immunomodulatory effects (attenuated MDSC suppression, alleviated T-cell exhaustion) remains indirect and speculative, requiring further mechanistic validation through Transwell co-culture experiments. Furthermore, the present study does not delineate the individual contributions of distinct bioactive components in SSG to the overall immunomodulatory effects. As a multi-component herbal preparation, SSG may act through combined and synergistic interactions among its constituents, rather than a single key compound. Future studies using isolated monomers or component-targeted modulation will be necessary to identify the main active ingredients and their individual or cooperative roles. Additionally, the LLC homologous mouse tumor model cannot fully replicate the heterogeneity characteristics of human LUAD, which may to some extent affect the clinical application of the research conclusions. Based on this, subsequent studies will introduce patient-derived xenograft models (PDX) and 3D organoid culture systems to further verify the immune regulatory effect and therapeutic potential of SSG in tumors with human LUAD heterogeneity characteristics, with the aim of promoting the transformation of research results into clinical practice.

## Conclusion

**Fig. 12:**
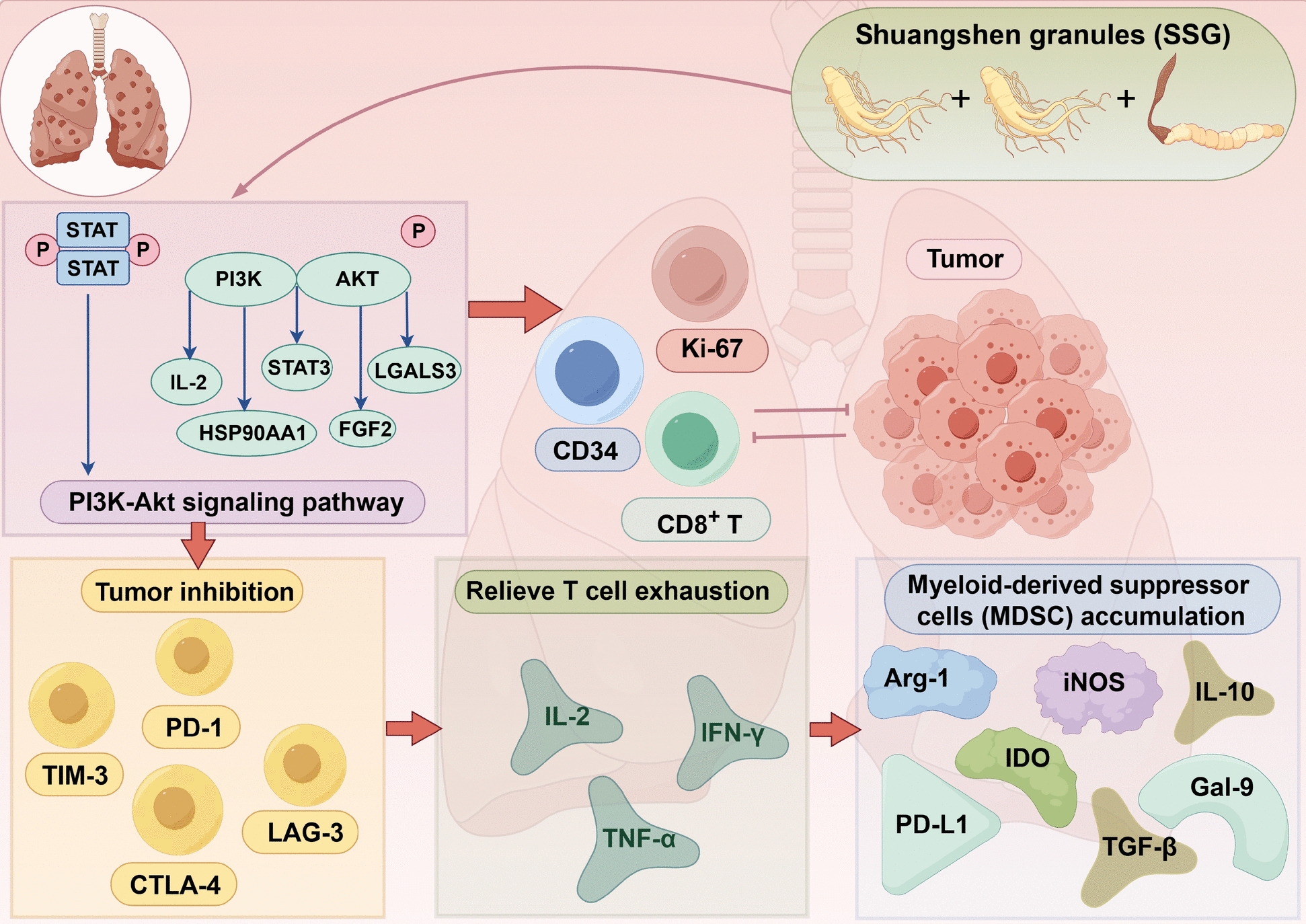
Mechanism by which SSG enhance anti-PD-1 therapy efficacy in LUAD through regulating the tumor immune microenvironment

In conclusion, this study provides experimental evidence that Shuangshen granules (SSG) enhance antitumor immunity in a LUAD model by modulating the tumor microenvironment. SSG reduced the frequency and suppressive mediators of MDSCs, alleviated T cell exhaustion, and improved the efficacy of anti-PD-1 checkpoint therapy. Based on the results of this study, we found that SSG can enhance the efficacy of anti-PD-1 therapy by regulating the tumor immune microenvironment, providing new insights for the treatment of LUAD (Fig. 12). These findings highlight the therapeutic potential of concurrently targeting myeloid immunosuppression and T cell dysfunction to overcome immunotherapy resistance. SSG represents a promising adjunctive strategy, though future studies are warranted to identify the key active components, clarify their synergistic or additive effects, optimize safe and effective dosing regimens, and further validate these immunological mechanisms in clinical settings.

## Supplementary Information

Supplementary Material 1.

Supplementary Material 2.

## Data Availability

The datasets used and/or investigated during the current study are available from the corresponding author upon reasonable request.

## Abbreviations

Arg-1: Arginase-1
CTLA-4: Cytotoxic T lymphocyte-associated antigen-4
ELISA: enzyme-linked immunosorbent assay
FBS: Fetal bovine serum
Gal-9: Galectin-9
GO: Gene Ontology
HE: Hematoxylin and eosin
IDO: Indoleamine 2,3-dioxygenase
iNOS: Inducible Nitric Oxide Synthase
IHC: Immunohistochemistry
IL-2: Interleukin-2
IFN-γ: Interferon-γ
IL-10: Interleukin-10
ICIs: Immune checkpoint inhibitors
KEGG: Kyoto Encyclopedia of Genes and Genomes
LLC: Lewis lung carcinoma
LAG-3: Lymphocyte Actuvation Gene-3
LUAD: Lung adenocarcinoma
MDSC: Myeloid-derived suppressor cells
NSCLC: Non-small-cell lung cancer
PD-1: Programmed cell death 1
PD-L1: Programmed cell death ligand 1
Rt-qPCR: Reverse transcription-quantitative polymerase chain reaction
SSG: Shuangshen granules
SSG-L: Low-dose SSG
SSG-M: Medium-dose SSG
SSG-H: High-dose SSG
TCM: Traditional Chinese medicine
TIM-3: T cell immunoglobulin domain and mucin domain-3
TNF-α: Tumor necrosis factor alpha
TGF-β: Transforming growth factor beta
TME: Immunosuppressive tumor microenvironment
TRMs: Tissue-resident memory T cells
