# Supplementary figures and images for: Shuangshen granules enhance anti-PD1 therapy effectiveness in lung adenocarcinoma by modulating myeloid-derived suppressor cell-induced T cell exhaustion
Source: Chin Med. 2026 May 6;21:126. doi: 10.1186/s13020-026-01391-3 (PMC13147628; doi:10.1186/s13020-026-01391-3)

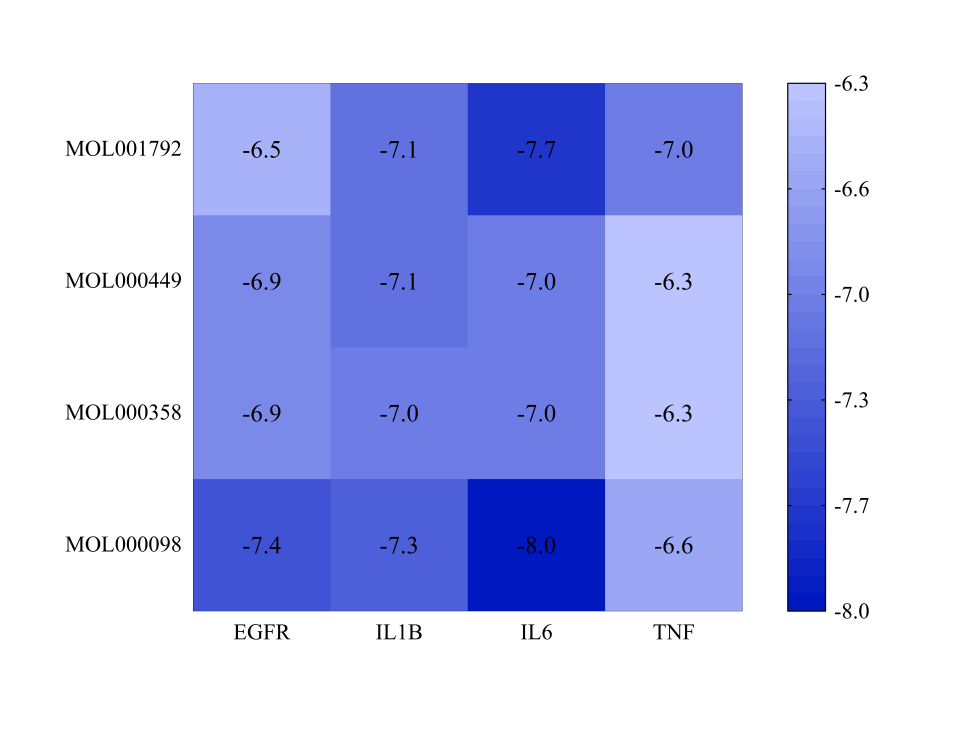


Supplementary Figure Heatmap of binding energy

Supplement: Supplementary file 1 — Supplementary Material 1. [file 13020_2026_1391_MOESM1_ESM.docx]

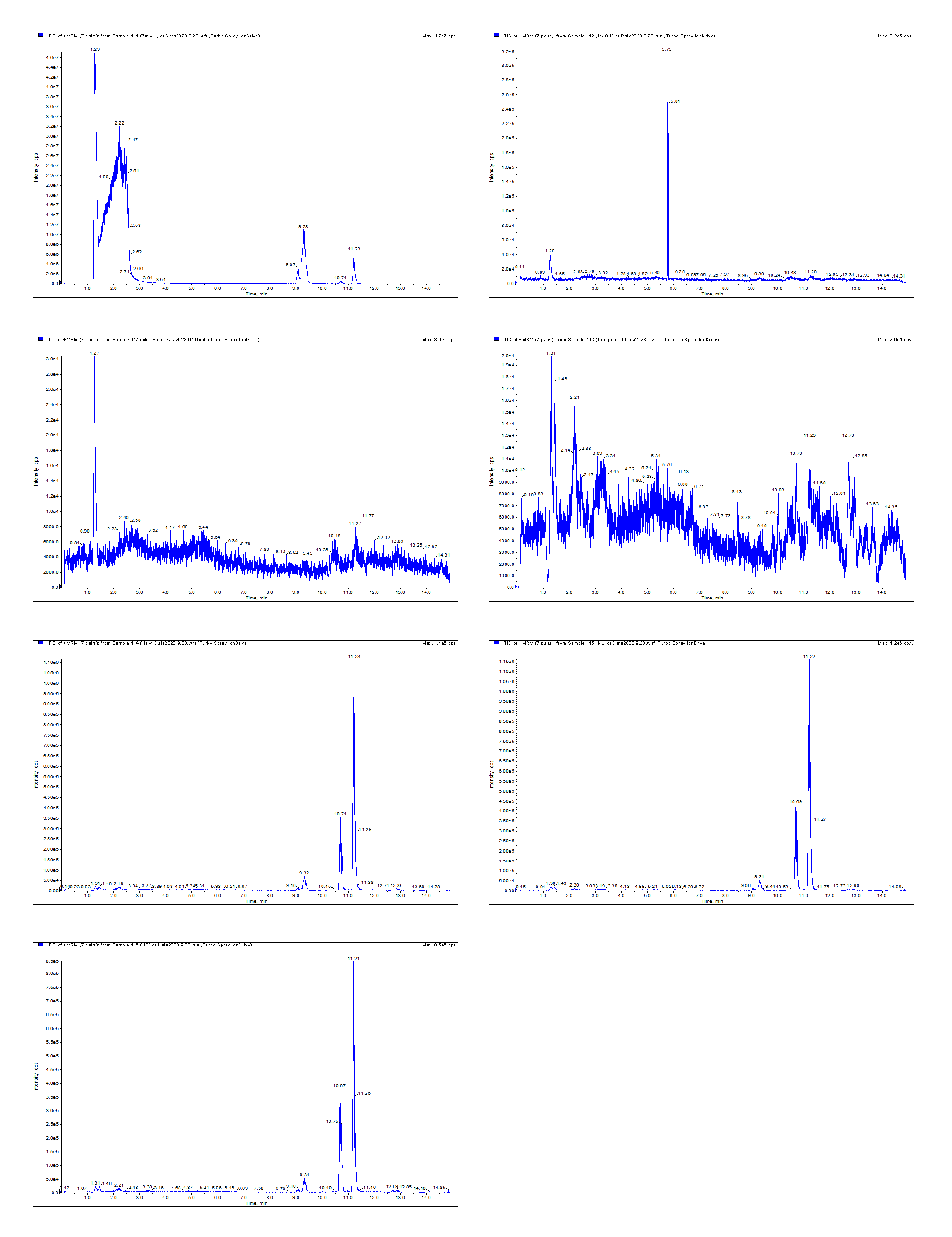

Supplement: Supplementary file 2 — Supplementary Material 2. [file 13020_2026_1391_MOESM2_ESM.tif]
